# Phytochemical Study of the Ecuadorian Species *Lepechinia mutica* (Benth.) Epling and High Antifungal Activity of Carnosol against *Pyricularia oryzae*

**DOI:** 10.3390/ph11020033

**Published:** 2018-04-19

**Authors:** Jorge Ramírez, Gianluca Gilardoni, Erika Ramón, Solveig Tosi, Anna Maria Picco, Carlo Bicchi, Giovanni Vidari

**Affiliations:** 1 Departamento de Química y Ciencias Exactas, Universidad Técnica Particular de Loja (UTPL), Calle M. Champagnat s/n, 1101608 Loja, Ecuador; gianluca.gilardoni@gmail.com (G.G.); erika11116@gmail.com (E.R.); 2 Dipartimento di Scienza della Terra e dell’Ambiente, Università degli Studi di Pavia, Via S. Epifanio 14, 27100 Pavia, Italy; solveig.tosi@unipv.it (S.T.); annamaria.picco@unipv.it (A.M.P.); 3 Dipartimento di Scienza e Tecnologia del Farmaco, Università degli Studi di Torino, Via P. Giuria 9, 10125 Torino, Italy; carlo.bicchi@unito.it; 4 Dipartimento di Chimica, Università degli Studi di Pavia, Via T. Taramelli 10, 27100 Pavia, Italy; cistre@unipv.it

**Keywords:** *Lepechinia mutica*, Lamiaceae, carnosol, secondary metabolites, essential oil, Ecuador, *Pyricularia oryzae*

## Abstract

The plant *Lepechinia mutica* (Benth.) Epling (family Lamiaceae) is endemic to Ecuador. In the present study, we report some major non-volatile secondary metabolites from the leaves and the chemistry of the essential oil distilled from the flowers. The main identified compounds were carnosol, viridiflorol, ursolic acid, oleanolic acid, chrysothol, and 5-hydroxy-4′,7-dimethoxy flavone. Their structures were determined by X-ray diffraction and NMR and MS techniques. The essential oil showed a chemical composition similar to that distilled from the leaves, but with some qualitative and quantitative differences regarding several minor compounds. The main constituents (>4%) were: δ-3-carene (24.23%), eudesm-7(11)-en-4-ol (13.02%), thujopsan-2-α-ol (11.90%), β-pinene (7.96%), valerianol (5.19%), and co-eluting limonene and β-phellandrene (4.47%). The volatile fraction was also submitted to enantioselective analysis on a β-cyclodextrin column, obtaining the separation and identification of the enantiomers for α-thujene, β-pinene, sabinene, α-phellandrene, limonene and β-phellandrene. Furthermore, the anti-fungal activity of non-volatile secondary metabolites was tested in vitro, with carnosol resulting in being very active against the “blast disease” caused by the fungus *Pyricularia oryzae*.

## 1. Introduction

The family Lamiaceae comprises approximately 224 genera and more than 5600 species distributed all over the world. One of these genera is *Lepechinia* Willd., consisting of 43 species growing from Northern California, in Western USA, to Central Argentina, in South America [1,2,3,4]. The flora of Ecuador contains 21 genera of Lamiaceae, for a total number of 135 species, 33 of which are endemic. In particular, the genus *Lepechinia* Willd. includes nine species, among which four are endemic to Ecuador [2,5]. *Lepechinia* species are perennial herbs to shrubs, rarely gynodioecious or dioecious, often aromatic. Leaves are entire to toothed, often rugose; inflorescences are terminal and often axillary [2,5]. Several *Lepechinia* spp. are valued in the horticultural trade, and indigenous peoples of North and South America commonly use *Lepechinia* plants for medicinal and antiseptic purposes. Plants of this genus are used in folk medicine for the treatment of uterine tumors, stomach ailments, diabetes mellitus control and diarrhea treatment. In particular, the leaves of *L. mutica* are used to treat headache and nervous affections [1,3,6,7], whereas the *L. caulescens* extract has been patented as a cosmetic constituent [8].

Fungal infections or mycoses are common public health problems, ranging from superficial to deep infections. Superficial mycoses sometimes reach high endemic levels, especially in tropical areas where dermatophyte fungi are usually the principal infection factor [9,10]. Indeed, it is often argued that dermatophyte infections are the most common human infection in the world (not just the most common fungal infection). On the other hand, rice blast disease, caused by *Pyricularia oryzae* Cavara (anamorph of *Magnaporthe oryzae* B. Couch sp. nov.) [11], is a severe hemibiotrophic pathogen of rice (*Oryza sativa* L.). This pathogen may destroy wide extensions of rice cultures, reaching up to 50−70% of a whole regional production. It has been calculated that the amount of rice annually destroyed by *P. oryzae* could feed more than 60 million people [12,13].

This fungus can cause two symptoms: leaf blast and neck blast [14]. The leaf blast is characterized by white to gray green lesions or spots with darker borders appearing on the leaves. The old lesions are elliptical or spindle-shaped and whitish to gray with necrotic border. The neck blast is characterized by dark brown lesions on the base of the panicle neck, so that it cannot support the panicle. *P. oryzae* can infect the rice plant at various growth stages [15]. Our research group is interested in the discovery of new antidermatophyte substances from natural resources [16] and new agents against plant pathogenic fungi. The aim of this research was the isolation and identification of antifungal compounds from *L. mutica*. In order to accomplish this task, the dermatophyte *Microsporum canis* and the phytopatogen *P. oryzae* were selected as targets for the in vitro antifungal assay of our samples. Up to now, the antifungal activity of this plant has been described only for the essential oil distilled from leaves [17,18]. To the best of the authors’ knowledge, the phytochemistry and the biological activity of *L. mutica* non-volatile fraction have not been described so far.

## 2. Results

### 2.1. Non-Volatile Fraction

**Figure 1:**
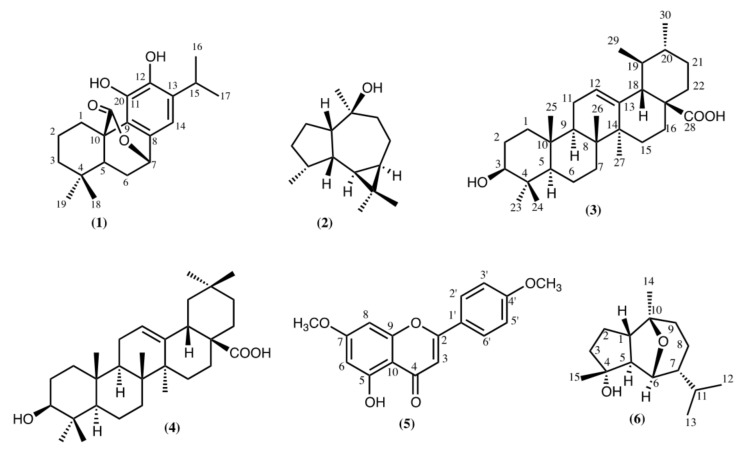
Compounds isolated from the leaves of *Lepechinia mutica.*

Six known compounds were identified after the fractionation of the ethyl acetate (EtOAc) extract, obtained from leaves of *L. mutica*, namely the phenolic diterpene, carnosol (**1**), the sesquiterpene alcohol viridiflorol (**2**), the pentacyclic triterpene acids, ursolic acid (**3**) and oleanolic acid (**4**), the flavonoid 5-hydroxy-4′,7-dimethoxy flavone (**5**), and the sesquiterpene alcohol chrysothol (**6**) (Figure 1). The presence of compounds **1**–**6** in *L. mutica* is reported here for the first time.

The structure of carnosol (**1**) was established by single crystal X-ray diffraction analysis. The crystallographic data were the same as those reported in the literature [19]: orthorhombic crystal; P21212; a = 15.762 (1), b = 13.757 (1), c = 7.7747 (7) Å, Z = 4, V = 1688.2 Å3, and R = 0.031. The structure elucidation was also supported by the comparison of the ^1^H NMR, ^13^C NMR, and ESI-MS data with those in the literature [20,21]. The ^13^C NMR and DEPT spectra indicated the presence of four methyls, four methylenes, two methines, one oxymethine, six aromatic carbons, two quaternary carbons and one ester carbon [22,23]. The aromatic ring must be penta-substituted and one substituent was an isopropyl group, according to the ^1^H NMR spectrum [20].

The aromadendrene sesquiterpene alcohol viridiflorol (**2**) was identified by comparison of the ^1^H NMR, ^13^C NMR, and ESI-MS data with those reported in the literature [24,25,26,27].

The pentacyclic triterpenoid acids ursolic acid (**3**) and the isomeric oleanolic acid (**4**) are widely occurring in plants in both free and glycosidic forms (saponins) [28]. They were identified by comparison of the ^1^H NMR, ^13^C NMR, and ESI-MS data with the literature [29,30,31,32].

5-Hydroxy-4′,7-dimethoxy flavone (**5**) was identified by comparison of the ^1^H NMR, ^13^C NMR, and ESI-MS data with those reported in the literature [33,34,35]. The ^1^H NMR spectrum clearly indicated that compound **5** was a flavone containing a free phenolic OH group and two methoxy groups. The one-proton singlet at δ 13.60 in the ^1^H NMR spectrum, exchangeable with D_2_O and assignable to a phenolic proton bridged to the carbonyl group, suggested a free phenolic OH group in position C-5 [35].

The oxygenated sesquiterpenoid chrysothol (**6**) was identified by comparison of the ^1^H NMR, ^13^C NMR, and ESI-MS data with those in the literature [36,37,38]. The ^1^H and ^13^C NMR spectra in CDCl_3_ showed signals of a guaiane sesquiterpenoid skeleton. The ^1^H NMR spectrum displayed a broad doublet at δ 3.94 (1H, *J* = 4.0 Hz, H-6), a multiplet at δ 2.26 for two protons (H-1 and H-5), and a multiplet at δ 2.07 (1H, H-3a). Additionally, signals assignable to the two secondary methyl groups of an isopropyl unit, at δ 0.87 (6H, *J* = 7.0 Hz, H-12 and H-13), and to two tertiary methyls at δ 1.12 and 1.34 (H-14 and H-15), respectively [34,39], were found. The ^13^C NMR and DEPT spectra data revealed 15 carbon signals assignable to four methyls, four methylenes, five methines and two quaternary carbons. Three oxygenated carbons were observed at δ 76.1 (d), 74.7 (s), and 74.6 (s) and the other 12 signals were for aliphatic carbons. All spectra data are available as supplementary material.

### 2.2. Chemical Analysis of the Volatile Fraction from Flowers

The flowers of *L. mutica* provided an essential oil in a yield of 0.317 ± 0.096% (*w*/*w*), referred to fresh plant material. The relative density was *d*^20^ = 0.8526 ± 0.0127, and the refractive index *n*^20^ = 1.4906 ± 0.0015. The specific rotation was [α]_D_^20^ = −6.0 (c 0.08 in CH_2_Cl_2_). The qualitative and percent compositions are reported in Table 1. About 93.9% of the volatile components have been identified and most of them quantified (see Section 4.5 and Section 4.6, respectively). All constituents with an abundance <0.1% were considered as trace. The significant difference between calculated and reference linear retention indices (LRIs) [40] is within the experimental error. It can be due to different analysis conditions, carried out with a more recent instrumentation.

### 2.3. Enantiomeric Analysis of the Volatile Fraction from the Flowers

**Figure 2:**
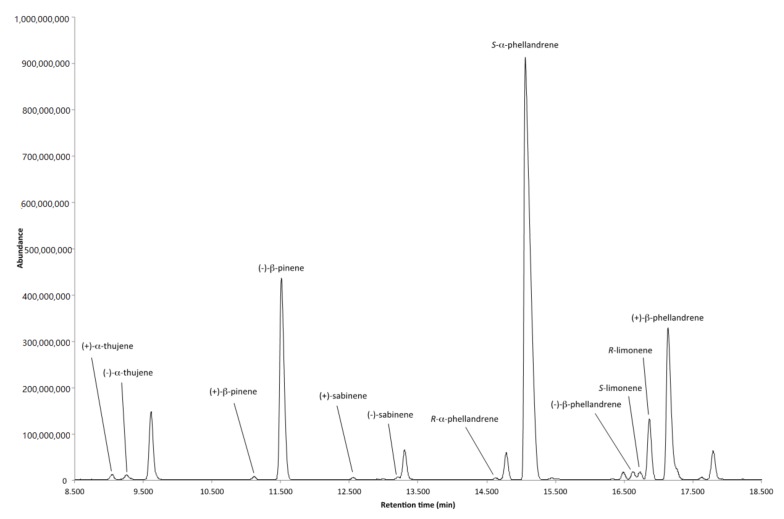
Chromatogram of enantiomers separated by enantioselective GC.

The results of the GC-MS analysis on a β-cyclodextrin enantioselective column are reported in Table 2, including both the enantiomeric distribution and the enantiomeric excess (*ee*) for each enantiomeric couple. The chromatogram of the enantioselective analysis is shown in Figure 2; only the enantiomeric couples that underwent separation have been reported. It is worthy of note that most of the detected enantiomers were baseline separated.

### 2.4. Biological Activity

Antifungal activities were determined for carnosol (**1**) and the results are shown in Table 3. Carnosol (**1**) showed a potent antifungal activity against the dermatophyte *Mircrosporum canis* (CBS 136538) and against *Pyricularia oryzae* (LM 120). The minimum inhibitory concentration (MIC) and minimum fungicidal concentration (MFC) values (mg/mL) determined for carnosol against *P. oryzae* are very close to those of the reference well-known pesticide flutriafol (0.01 mg/mL). Although this result does not validate a property of the plant extract, it constitutes a new biological property described for compound **1**. These results clearly show that the activity of carnosol (**1**) is similar to that of flutriafol on *Pyricularia oryzae* but not competitive to that of intraconazol on *Mircrosporum canis*. More experiments are under way in this respect.

## 3. Discussion

### 3.1. Non-Volatile Fraction

Over 71 years ago, a bitter principle was isolated from sage, *Salvia carnosa* Dougl. The natural substance, having the formula C_20_H_26_O_4_, was named carnosol and noted to be a diphenolic ester, containing a hydrophenanthrene moiety, presumably identical to a compound also obtained from *S. officinalis* L. Indeed, the correct structure was eventually deduced from chemical studies mainly performed on a sample isolated from *Rosmarinus officinalis*. It was also showed that carnosol was identical with pikrosalvin (picrosalvin) isolated from *S. officinalis* L. [23,39]. Other sources of carnosol are *S. triloba* L., *Sphacele eremophila* Boiss., *S. pachyphyla*, *S. clevelandii*, and *Lepechinia hastate* [39,42]. Carnosol was also isolated, as the main diterpene constituent, from the aerial parts of *Sphacele chamaedryoides*, and it was determined to be more cytotoxic against gastric tumour cells AGS than normal fibroblasts [43]. Moreover, it showed a significant activity against the gram (+) bacteria *Staphylococcus aureus*, *Bacillus subtilis*, *Escherichia coli*, and *Candida albicans* [44].

Carnosol showed strong inhibitory effect on the EBV-EA induction and exhibited a remarkable effect on mouse skin tumor promotion in an in vivo two-stage carcinogenesis model. Carnosol has also been shown to be partly responsible for the antioxidant and anti-tumorogenesis properties of rosemary (*Rosmarinus officinalis* L.). Recently, carnosol has been proved to be efficacious in the chemoprevention and/or chemotherapy of hormone-responsive cancers [45,46,47].

The cytotoxicity activity of carnosol (**1**) has been determined in vitro against A-549 (human lung carcinoma), HeLa (human carcinoma of the cervix), HEp-2 (human carcinoma of the larynx), and MCF-7 (human breast adenocarcinoma) cell lines, and against a *Vero* (African green monkey kidney) cell line [46].

Viridiflorol (**2**) was previously isolated, as the major component, from the essential oils obtained from *Cistus ladaniferus* L. [27], “niaouli” (*Melaleuca viridiflora*, *M. quinquenervia*) [24,48], and the liverwort *Bazzania trilobata* L. [49]. It has also been synthesized via (-)-alloaromadendrene [26]. Viridiflorol (**2**) showed antifungal activity against *C. cucumerinum* and *P. oryzae* [26,49].

Oleanolic acid (**4**) was isolated from more than 120 plant species, as it results from a partial survey about its occurrence in plants used in folk medicine, and its biological activity. As a typical example, both oleanolic acid (**4**) and ursolic acid (**3**) are present in *Sambucus chinesis* Lindl. This plant is used in folk medicine to treat inflammatory disorders and acute hepatitis. Compounds **3** and **4** were identified as the metabolite responsible for this activity [29]. Ursolic acid is chemically and pharmacologically similar to oleanolic acid. The multiple uses of oleanolic or ursolic acid containing plants in folk medicines are due to anti-inflammatory, hepatoprotective, analgesic, cardiotonic, sedative and tonic effects, among others [29].

Ursolic acid (**3**) was identified as an active hepatoprotective component in the preparation of *Sambucus chinesis* Lindl., *Solanum incanum* L., *Tripterospermum taiwanense*, and *Eucalyptus hybrid*. In addition to its protective effects against CCl_4_-induced liver injury, ursolic acid (**3**) also shows protective properties against D-galactosamine-induced liver injury in rats, and preventive effects against acetaminophen-induced cholestasis [28].

Ursolic acid (**3**) and oleanolic acid (**4**) was also isolated from *Lepechinia caulescens*. The vascular activity of ursolic acid (**3**) depended on the presence of a functional endothelium and possible NO release [29].

Flavonoid **5** was previously isolated from the aerial parts of *Sphacele chamaedryoides* [41], *Ballota inaequidens* [34], *Salvia texana* [35], *Artemisia pontica* [50], and *Combretum erythohyllum* [51]. It was also identified and quantified in *Kaempferia parviflora* and *Cistus libanotis* [52,53]. Compound **5** showed good activity against *Vibrio cholerae* and *Enterococcus faecalis*, with MIC values in the range of 25–50 μg/mL. Furthermore, it is also potentially toxic to human cells [51], and possesses high anti-allergenic activity against antigen-induced β-hexosaminidase release as a marker of degranulation in RBL-2H3 cells, with an IC_50_ of 26 μM [52].

Chrysothol (**6**) was isolated before from *Chrysothamnus viscidiflorus* (Asteraeceae) and *Fagonia boveana* [38], and showed important anti-cancer activity against human breast cancer cells [36].

### 3.2. Volatile Fraction

The essential oil distilled from flowers of *L. mutica* is composed of monoterpenoid and sesquiterpenoid compounds (55.6% and 44.4% respectively). The main constituents (>4%) were: δ-3-carene (24.2%), eudesm-7(11)-en-4-ol (13.0%), thujopsan-2-α-ol (11.9%), β-pinene (8.0%), valerianol (5.2%), and co-eluting monoterpenoids limonene and β-phellandrene (4.5%). Since major constituents of the essential oil distilled from flowers are the same as the one distilled from leaves, the two chromatograms result in being qualitatively quite similar. However, quantitative analysis demonstrates that the overall composition of the two volatile fractions is actually different. In Table 1, the quantitative chemical composition of the two essential oils, expressed as percent abundance, is reported. Aliphatic monoterpens and sesquiterpens are the families showing the greatest difference, passing respectively from 24.5% to 48.9% in flowers and from 9.9% to 36.0% in leaves.

## 4. Materials and Methods

### 4.1. Instruments and Disposables

Solvents were reagent grade or HPLC grade and were purchased from Sigma-Aldrich (St. Louis, MO, USA). Low pressure preparative column chromatography was carried out on Merck LiChroprep RP-18 (Darmstadt, Germany) (25–40 μm) or Merck silica gel (230–400 mesh), in reversed and normal phase column chromatography, respectively. Medium pressure flash-chromatography was carried out on Biotage^®^ Isolera™ Spektra equipment (Biotage AB, Uppsala, Sweden), equipped with a UV detector (205 and 254 nm) and a C-18 Cartridge (KP-18-HS), 100 g, operating with an eluent flow of 30 mL/min. TLC was performed over Merck F_254_ glass plates (RP-18) or aluminum supported silica sheets (0.25 mm), purchased from Sigma-Aldrich. Spots were detected under UV light (254 and 366 nm) and, additionally, stained by exposure to a 0.5% solution of vanillin in H_2_SO_4_-EtOH (4:1), followed by heating at 100 °C. The SPE columns, used for chlorophyll removal, were Discovery DSC-18 (Sigma-Aldrich) 60 mL tubes, containing 10 g of C-18 reversed phase. Optical rotations were measured on a Perkin-Elmer 241 polarimeter (Waltham, MA, USA) (concentration expressed in g/mL). Melting points were measured with a Thermo Scientific Fisher–Johns hot-plate instrument. Refractive indices were measured with an Abbe refractometer (Boeco Germany, Hamburg, Germany). LC-MS spectra were obtained with a Thermo Scientific LTQ XL Linear ION Trap mass spectrometer, equipped with an ESI source. X-ray crystallographic analysis was performed at the “Centro Grandi Strumenti (CGS)” of the University of Pavia. NMR spectra were recorded on an NMR Varian 400 MHz spectrometer or, alternatively, on Bruker 300 MHz and 200 MHz instruments. Chemical shifts (δ are given in ppm and coupling constants (*J*) in Hz. GC-MS and GC-FID analyses were performed with an Agilent Technologies 6890N GC, coupled to a mass spectrometry detector model 5973 *inert*, and to a flame ionization detector. In qualitative and quantitative GC analyses, a J&W DB-5 capillary column (30 m × 0.25 mm × 0.15 μm), purchased from Agilent Technologies, was used. GC enantioselective analysis was performed with a cyclodextrin-based capillary column (30% 2,3-diethyl-6-*tert*-butyldimethylsilyl-β-CDX, 25 m × 0.25 mm × 0.25 μm), purchased from Mega (Legnano, Italy).

### 4.2. Plant Material

The collection of *L. mutica*, authorized by the Ministry of Environment of Ecuador (MAE), with authorization no. 001-IC-FLO-DBAP-VS-DRLZCH-MA, was performed in the canton Quilanga, Loja province, Ecuador, in September 2009. The botanical specimen was identified by Dr. Bolivar Merino at the herbarium of the “Universidad Nacional de Loja”. A voucher specimen, with number PPN-la-005, was deposited at the herbarium of the “Universidad Técnica Particular de Loja”. After collection, leaves and flowers were separated and submitted to different processes. After drying in the dark at 35 °C, the leaves were submitted to solvent extraction. Fresh leaves and flowers were both steam distilled to obtain the essential oils. A quite complete study of the essential oil from leaves has recently been published [17].

### 4.3. Extraction and Purification of Non-Volatile Metabolites

The extract was obtained from 250 g of dried leaves of *L. mutica*, using EtOAc as the extraction solvent. The process was carried out statically, at room temperature, for three consecutive times, one hour each. After solvent evaporation, 24.1 g of the EtOAc extract were obtained (yield: 9.6%). The extract underwent subsequent removal of chlorophylls by filtration through a C-18 SPE column; elution was done with MeOH-H_2_O, 9:1 *v*/*v*, followed by 100% acetone. After chlorophyll removal and solvent evaporation chlorophyll, a free EtOAc extract (13.8 g) was obtained. In addition, 1 g of this extract was fractionated by medium-pressure flash chromatography on a C-18 cartridge, containing 100 g of stationary phase, with an elution gradient from methanol/water (2:1) to 100% methanol. The eluent flow was set at 30 mL/min, giving 19 main fractions. Fraction #11 (511.5 mg) was subjected to normal phase (silica gel) column chromatography. Elution with a mixture of hexane/EtOAc (97:3) gave 38.5 mg of carnosol (**1**). Fraction #12 and fraction #13 (58.6 mg) were collected together and submitted to silica gel column chromatography. From the elution with a mixture of hexane/EtOAc (95:5), 10.0 mg of viridiflorol (**2**) was obtained. Fraction #18 (79.9 mg) was submitted to C-18 reversed phase column chromatography. Elution with a mixture of MeOH/H_2_O (95:5) afforded 14.2 mg of ursolic acid (**3**), and 23.5 mg of oleanolic acid (**4**). A second amount of the EtOAc chlorophyll-free extract (11 g) was fractionated by preparative column chromatography on silica gel in order to get larger amounts of metabolites from *L. mutica*. Elution with an increasing polarity gradient of a hexane/EtOAc mixture afforded 16 main fractions. Fraction #11 (4.9 g) was subjected to silica gel column chromatography. Elution with an increasing polarity gradient of a hexane/EtOAc mixture gave 11.9 mg of 5-hydroxy-4′,7-dimethoxy flavone (**5**), and 127.7 mg of carnosol (**1**). Collected fractions #13 and #14 (2.4 g) were submitted to silica gel column chromatography. Elution with an increasing polarity gradient of a hexane/EtOAc mixture gave 11 main fractions (F38-F49). Fraction #44 (158.3 mg) was also submitted to silica gel column chromatography. Elution with an increasing polarity gradient of a dichloromethane/EtOAc mixture afforded 77.7 mg of isolated chrysothol (**6**).

### 4.4. Distillation of the Volatile Fraction

Fresh flowers of *L. mutica* (5 kg) were divided into four equal amounts and steam was distilled in four stainless steel Clevenger-type apparatus for 4 h. After distillation, each organic layer was separated from the aqueous phase, dried over anhydrous sodium sulfate and weighted. For each distilled fraction, yield, relative density, refractive index and specific rotation were measured. The flowers were handled and distilled in the same way described for leaves [17].

### 4.5. Qualitative Analysis of the Essential Oil

The qualitative analysis of the essential oil was performed by GC-MS, injecting 1 μL of each distilled fraction, 1% (*v*/*v*) diluted in cyclohexane. The injector was kept at 220 °C, operating in split mode with a split ratio of 40:1. The carrier gas (He) was set at a constant flow of 1 mL/min. The analysis was performed in thermal gradient conditions, with the following temperature program: 60 °C for 5 min, increased to 110 °C at a rate of 5 °C/min, then to 148 °C at a rate of 2 °C/min. and to 250 °C at a rate of 20 °C/min, and then held at 250 °C for 2.4 min. The MS was operated in a SCAN mode, with a scan rate of 2 scan/s within a mass range of 40–350 *m*/*z* at 70 eV. For each chromatographic peak, the corresponding linear retention index (LRI) was calculated, according to Van den Dool and Kratz [41], with reference to a mixture of a homologous series of *n*-alkanes, from nonane to heptadecane. The constituents of the essential oil were identified, by comparing their LRIs and EI-MS spectra with data present in literature [40]. The identification was considered as acceptable in a range of ±13 units of LRI values, according to the injection of some standard compounds belonging to the different terpenic families.

### 4.6. Quantitative Analysis of the Essential Oil

The quantitative analysis of the essential oil was performed by GC-FID, with the same instrumental configuration and method of the qualitative analysis. The constituents were quantified by external calibration, using *n*-nonane as the internal standard. According to the literature [54], the response factor in FID detection is almost constant and close to value 1 within isomeric compounds, allowing the use of a single standard to quantify a family of analytes, all characterized by the same molecular formula. The same considerations are reported in reference [55]. Therefore, an isomer was used to quantify isomeric metabolites; if an isomer was not available, a structurally closely related terpene was selected. Hence, the following terpenoids (purity > 98%) were used as calibration standards: limonene for aliphatic monoterpene hydrocarbons (R^2^ = 0.9962), *p*-cymene for aromatic monoterpene hydrocarbons (R^2^ = 0.9986), linalool for monoterpene alcohols (R^2^ = 0.9956), carvone for monoterpene ketones (R^2^ = 0.9958), cedrene for sesquiterpene hydrocarbons (R^2^ = 0.9998), and nerolidol for sesquiterpene alcohols (R^2^ = 0.9997). All calibration curves were built on six points. Quantitative results were reported as the main values and standard deviations of three replicates for each distillation.

### 4.7. Enantioselective Analysis of the Essential Oil

The enantioselective analysis was performed by GC-MS, under the same conditions reported above except for the oven temperature that was set according to this program: initial temperature hold at 60 °C for 2 min, then increased to 220 °C at a rate of 2 °C/min, and then held at 220 °C for 2 min. The elution order of the separated enantiomers was determined by injection of enantiomerically pure standards, available in the laboratory of one of the authors (C.B.). LRIs were calculated as in qualitative analysis, according to Van den Dool and Kratz (see Section 4.5).

### 4.8. Antifungal Activity

Antifungal activities were expressed as the minimum inhibitory concentration (MIC) and minimum fungicidal concentration (MFC). MIC and MFC values were measured using a stereoscope and were determined for two strains of *Microsporum canis* (CBS 136538, from CBS-KNAW culture collection, NL), a human dermatophyte fungus, and a *Pyricularia oryzae* (LM120 strain, isolated by S.T. and A.M.P. from leaf lesions of Italian rice varieties), a plant pathogenic fungus. The compounds were dissolved in dimethylsulfoxide (DMSO), 1% *v*/*v*, in the liquid medium culture Sabouraud. The tested concentrations (expressed as mg/mL) were: 0.1000, 0.0500, 0.0250, 0.0125, and 0.0060.

The commercial well-known pesticide flutriafol, containing the antifungal compound (*R*,*S*)-2,4-difluoro(1H-1,2,4-triazol-1-ylmethyl)benzhydryl alcohol, was used as the positive control against *P. oryzae*, and was tested at the following concentrations: 0.2000, 0.1000, 0.0500, 0.0250, 0.0125, and 0.0060 mg/mL. Itraconazole was used as the positive control to test carnosol against *Microsporum canis*, using the ATB FUNGUS 3 strip system (BioMerieux, La Balme-les Grottes, France). The following concentrations were used (µg/mL): 4.000, 2.000, 1.000, 0.500, 0.250, and 0.125.

The activities of the reference pesticides are assumed as criteria to evaluate the carnosol (**1**) antifungal properties.

## 5. Conclusions

In this work, the results of the first chemical investigation on both non-volatile and volatile secondary metabolites from the leaves and flowers, respectively, of *L. mutica* are reported. Carnosol (**1**), ursolic acid (**3**), oleanolic acid (**4**), viridoflorol (**2**), chrysothol (**6**), and 5-hydroxy-4′,7-dimethoxy flavone (**5**) have been isolated from the leaves. Furthermore, the chemical analysis and the enantiomeric recognition of the essential oil distilled from the flowers of *L. mutica* are described. The qualitative compositions of the essential oils from the flowers and leaves of *L. mutica* have also been compared. δ-3-Carene, eudesm-7(11)-en-4-ol, thujopsan-2-α-ol, β-pinene, valerianol, limonene, and β-phellandrene were the major components of both oils, while at least 16 minor compounds can be considered qualitatively different in the two volatile fractions.

The known phenolic diterpene lactone carnosol (**1**) was the main secondary metabolite isolated from *L. mutica*. It showed a powerful antifungal activity against the plant pathogenic fungus *P. oryzae* and an important antifungal activity against the dermatophyte fungus *Microsporum canis*.

## Figures and Tables

**Table 1:** Chemical composition of the essential oils from flowers and leaves of *Lepechinia mutica* on a DB-5 capillary column.

Calculated LRI ^a^	Reference LRI ^b^	Compounds	Flowers		Leaves [17]	
			%	σ	%	σ
921	926	Tricyclene	**-**	**-**	Trace	-
924	924	Thujene <α->	Trace	-	Trace	-
931	932	Pinene <α->	2.68	0.95	1.23	0.89
946	949	Camphene	**-**	**-**	0.75	0.80
948	945	Fenchene <α->	Trace	-	**-**	**-**
971	969	Sabinene	Trace	-	0.24	0.15
974	983	Oct-3-en-1-ol	**-**	**-**	Trace	-
976	974	Pinene <β->	7.96	0.99	3.78	1.76
979	989	Hepten-2-ol <6-methyl-5->	0.32	0.12	**-**	**-**
984	979	Octanone <3->	0.16	0.17	**-**	**-**
988	988	Myrcene	1.51	0.47	0.52	0.28
998	-	Undetermined (MW 136)	0.13	0.14	**-**	**-**
1003	1003	Mentha-1(7),8-diene <*p*->	Trace	-	0.16	0.13
1006	1002	Phellandrene <α->	0.34	0.13	3.80	1.70
1008	1008	Carene <δ-3->	24.23	6,00	8.69	4.24
1016	1014	Terpinene <α->	Trace	-	0.11	0.07
1019	1020	Cymene <*p*->	1.97	0.50	0.10	0.06
1023	1022	Cymene <*o*->	2.04	0.52	**-**	**-**
1025	1023	Sylvestrene	**-**	**-**	0.29	0.18
1029	1024	Limonene	4.47	0.71	3.79	2.18
1029	1030	Phellandrene <β->				
1043	-	Undetermined (MW 98)	0.23	0.46	**-**	**-**
1045	1044	Ocimene <(*E*)-β->	0.35	0.42	**-**	**-**
1052	1054	Terpinene <γ->	Trace	-	0.23	0.12
1057	-	Undetermined (MW 136)	0.52	0.20	**-**	**-**
1065	1071	*cis*-Sabinene hydrate	**-**	**-**	Trace	-
1070	-	Undetermined (MW 136)	0.20	0.40	**-**	**-**
1080	1085	Mentha-2,4(8)-diene <*p*->	0.86	0.25	0.35	0.18
1084	1086	Terpinolene	1.78	0.40	0.60	0.33
1084	1086	*trans*-Linalool oxide	**-**	**-**	Trace	-
1088	1082	Cymenene <*m*->	1.97	0.55	**-**	**-**
1095	1102	Linalool	**-**	**-**	0.20	0.09
1108	-	Undetermined (MW 136)	3.26	0.71	**-**	**-**
1110	1109	Oct-1-en-3-yl acetate	**-**	**-**	1.37	0.60
1120	-	Undetermined (MW 136)	0.27	0.12	**-**	**-**
1124	1117	Sabina ketone <dehydro->	Trace	-	**-**	**-**
1141	1145	Camphor	**-**	**-**	Trace	-
1142	-	Undetermined (MW 136)	0.10	0.19	**-**	**-**
1165	1172	Borneol	**-**	**-**	0.25	0.05
1174	1180	4-Terpineol	**-**	**-**	0.14	0.02
1194	1186	Terpineol <α->	0.16	0.04	0.11	0.02
1283	1281	Isobornyl acetato	**-**	**-**	2.20	1.04
1284	1284	Bornyl acetate	Trace	-	**-**	**-**
1335	1328	Elemene <δ->	**-**	**-**	Trace	-
1345	1348	Cubebene <α->	0.22	0.07	0.57	0.08
1346	1342	Terpinyl acetate <α->				
1373	1373	Ylangene <α->	0.26	0.09	0.15	0.05
1374	1362	Isoledene	0.20	0.05		
1374	1367	Copaene <α->	**-**	**-**	1.46	0.23
1381	1387	Bourbonene <β->	0.20	0.05	0.47	0.25
1385	1382	Modheph-2-ene	0.21	0.05	**-**	**-**
1392	1387	Cubebene <β->	0.20	0.05	0.15	0.04
1395	1398	Cyperene	0.20	0.05	**-**	**-**
1404	1400	Sibirene	0.28	0.09	**-**	**-**
1407	1409	Gurjunene <α->	0.20	0.05	1.94	0.37
1407	1418	Longifolene	**-**	**-**	0.15	0.07
1417	1411	Funebrene <2-*epi*-β->	0.57	0.19	Trace	-
1417	1412	(*E*)-Caryophyllene	**-**	**-**	4.55	2.16
1424	1431	Copaene <β->	0.20	0.05	0.50	0.08
1427	1421	Duprezianene <β->	0.21	0.06	**-**	**-**
1431	1431	Gurjunene <β->	0.21	0.05	1.47	0.78
1435	1437	Guaiene <α->	0.25	0.07	**-**	**-**
1439	1449	Aromadendrene	**-**	**-**	0.56	0.10
1443	1442	Guaiadiene <6,9->	0.20	0.05	**-**	**-**
1446	1448	Muurola-3,5-diene <*cis*->	0.21	0.06	0.45	0.36
1453	1452	Humulene <α->	0.29	0.08	1.20	0.47
1457	1452	Clovene <α-*neo*->	0.22	0.06	**-**	**-**
1459	1464	Caryophyllene <9-*epi*-(*E*)->	0.21	0.06	**-**	**-**
1461	1452	*cis*-Cadina-1(6),4-diene	**-**	**-**	0.99	1.36
1469	1465	Muurola-4(14),5-diene <*cis*->	0.22	0.06	**-**	**-**
1471	1463	Dauca-5,8-diene	**-**	**-**	0.38	0.09
1472	1469	Acoradiene <β->	0.23	0.07	**-**	**-**
1475	1466	*trans*-Cadina-1(6),4-diene	**-**	**-**	0.99	0.12
1478	1479	Amorpha-4,7(11)-diene	0.21	0.06	0.15	0.07
1482	1478	Muurolene <γ->	0.21	0.06	0.92	0.23
1486	1489	Selinene <β->	0.23	0.06	**-**	**-**
1488	1485	Himachala-1,4-diene <11-αH->	0.23	0.06	**-**	**-**
1492	1481	*cis*-b-Guaiene	**-**	**-**	0.71	0.11
1493	1486	Bicyclogermacrene	**-**	**-**	4.62	0.58
1493	1489	*epi*-Cubebol				
1493	1489	Zingiberene <α->				
1493	1492	Selinene <δ->	0.24	0.07	0.81	0.08
1496	1493	Muurola-4(14),5-diene <*trans*->	0.25	0.07	**-**	**-**
1500	1505	Cuprenene <α->	0.20	0.05	**-**	**-**
1503	1505	Farnesene <(*E*,*E*)-α->	0.20	0.05	0.83	0.25
1510	1500	Muurolene <α->	0.32	0.11	0.91	0.17
1513	1505	Cadinene <γ->	**-**	**-**	2.86	0.37
1514	1508	Cubebol	**-**	**-**	0.36	0.21
1516	1511	Amorphene <δ->	0.44	0.19	**-**	**-**
1521	1512	*trans*-Calamenene	**-**	**-**	0.15	0.04
1522	1511	Cadinene <δ->	**-**	**-**	6.96	0.99
1529	1528	Zonarene	0.21	0.06	**-**	**-**
1533	1523	*trans*-Cadina-1,4-diene	**-**	**-**	0.37	0.10
1534	1537	Cadinene <α->	0.21	0.06	0.39	0.12
1538	1545	Selina-3,7(11)-diene	0.20	0.05	0.14	0.04
1548	-	Undetermined (MW 204)	0.21	0.07	**-**	**-**
1555	-	Undetermined (MW 204)	0.21	0.06	**-**	**-**
1559	1556	Dauca-4(11),7-diene <*trans*->	0.20	0.05	**-**	**-**
1567	1559	Germacrene B	0.21	0.05	0.18	0.06
1574	1567	Germacrene D-4-ol	**-**	**-**	1.46	0.40
1574	-	Undetermined (MW 204)	0.25	0.04	**-**	**-**
1579	-	Undetermined (MW 204)	0.21	0.05	**-**	**-**
1582	1569	Caryophyllene oxide	**-**	**-**	0.29	0.24
1583	-	Undetermined (MW 204)	0.21	0.05	**-**	**-**
1590	1584	Globulol	**-**	**-**	5.91	2.61
1591	1586	Thujopsan-2-α-ol	11.9	1.76	**-**	**-**
1592	1592	Viridiflorol	**-**	**-**	1.29	0.45
1601	1600	Guaiol	0.15	0.12	**-**	**-**
1612	-	Undetermined (MW 204)	0.22	0.06	**-**	**-**
1618	1617	1,10-di-*epi*-Cubenol	**-**	**-**	0.27	0.11
1618	1623	Junenol	**-**	**-**	1.39	0.42
1629	1622	Eudesmol <10-*epi*-γ->	0.83	0.10	0.54	0.15
1634	1630	Eudesmol <γ->	2.02	1.48	**-**	**-**
1636	1639	Acorenol <β->	**-**	**-**	0.47	0.81
1639	1632	Acorenol <α->	0.60	1.20	Trace	-
1642	1635	Cadin-4-en-7-ol <*cis*->	0.88	1.75	**-**	**-**
1649	1644	Eudesmol <β->	-	-	4.47	1.93
1652	1644	Eudesmol <α->				
1652	1646	Cadinol <α->				
1652	1656	Valerianol	5.19	0.66	-	-
1668	1658	Selin-11-en-4-α-ol	Trace	-	-	-
1688	1681	Shyobunol	-	-	10.80	5.91
1691	1700	Eudesm-7(11)-en-4-ol	13.02	4.25	-	-
Aliphatic monoterpene hydrocarbons			48.89	-	24.54	-
Aromatic monoterpene hydrocarbons			5.98	-	0.10	-
Monoterpene alcohols			0.48	-	0.70	-
Monoterpene ketones			0.16	-	Traces	-
Aliphatic esters			-	-	3.57	-
Aliphatic sesquiterpene hydrocarbons			9.86	-	35.98	-
Sesquiterpene alcohols			34.59	-	27.25	-
TOTAL			99.96	-	92.14	-

^a^ According to van den Dool and Kratz [41]. ^b^ According to Adams [40].

**Table 2:** Enantiomeric analysis of the essential oil from flowers of *Lepechinia mutica* on a 30% 2,3-diethyl-6-*tert*-butyldimethylsilyl-β-CDX column.

Enantiomers	Retention Time (min)	LRI	Enantiomeric Distribution (%)	*ee* (%)
(+)-α-thujene	9.05	924	44.19	11.62
(−)-α-thujene	9.26	928	55.81	
(+)-β-pinene	11.12	962	1.48	97.04
(−)-β-pinene	11.51	969	98.52	
(+)-sabinene	12.55	988	46.37	7.26
(−)-sabinene	13.21	1000	53.63	
(*R*)-α-phellandrene	14.64	1024	7.17	85.66
(*S*)-α-phellandrene	14.79	1027	92.83	
(*S*)-limonene	16.73	1059	34.19	31.62
(*R*)-limonene	16.87	1062	65.81	
(−)-β-phellandrene	16.63	1058	4.34	91.32
(+)-β-phellandrene	17.14	1066	95.66	

**Table 3:** MIC and MFC of carnosol against *M. canis* and *P. oryzae.*

Compound	*Microsporum canis* (CBS 136538)		*Pyricularia oryzae* (LM120)	
	MIC (mg/mL)	MFC (mg/mL)	MIC (mg/mL)	MFC (mg/mL)
Carnosol (**1**)	0.0250 < MIC ≤ 0.0500	MFC > 0.1000	0.0125 < MIC ≤ 0.0250	0.0500 < MFC ≤ 0.1000
Flutriafol	-	-	0.0100	0.0100
Intraconazol	0.0005	-	-	-

## Supplementary Materials

The following are available online at http://www.mdpi.com/1424-8247/11/2/33/s1.
