# Supplementary material for: Phytochemical Study of the Ecuadorian Species Lepechinia mutica (Benth.) Epling and High Antifungal Activity of Carnosol against Pyricularia oryzae
Source: Pharmaceuticals (Basel). 2018 Apr 19;11(2):33. doi: 10.3390/ph11020033 (PMC6027405; doi:10.3390/ph11020033)
Supplement: Supplementary file 1 [file pharmaceuticals-11-00033-s001.pdf]

Spectroscopic data.

Carnosol (1)

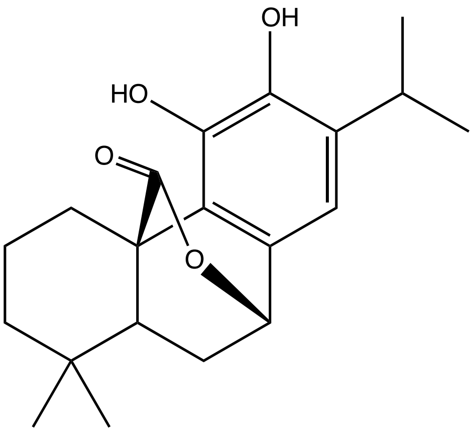

(1)

Colorless crystals, mp: 233-237ºC, $[\alpha]^{20}_{D}$= –51,9 (*c* 0.05 in $CHCl_3$). The molecular formula $C_{20}H_{26}O_4$ was established from the MS (EI^+) M^+ at *m/z* 331.42 $[M+H]^+$. ^{1}H-NMR (CD_3OD, 200 MHz) δ 6.69 (H, s, H-14), 5.44 (1H, dd, *J* = 3.8, 1.4 Hz, H-7), 3.28 (1H, sept, *J* = *6.8*, H-15), 2.65 (1H, dd, *J* = 14, 1.6 Hz, H-1α), 2.49 (1H, ddd, *J* = 14, 4.6 Hz, H-1β), 1.91 (1H, ddd, H-6α), 1.6 (1H, m, H-6β), 1.56 (1H, dd, *J* = 10.6, 5.4 Hz, H-5), 1.50 (1H, m, H-2β), 1.42 (1H, dd, *J* = 13, 1.4 Hz, H-3α), 1.21 (1H, ddd, *J* = 13.4, 3.2 Hz, H-3β), 1.10 (2×3H, d, *J* = 6.8, H_3-17, H_3-16), 0.87 (3H, s, H-18), 0.87 (3H, s, H-19).

Viridiflorol (2)

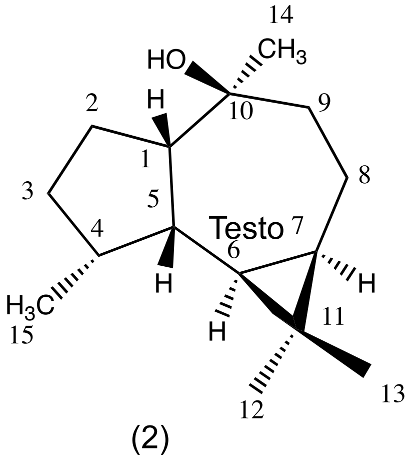

(2)

Colorless oil, $[\alpha]^{20}_{D}$= +11.70 (*c* 0.04 in CH_2Cl_2). The molecular formula $C_{15}H_{26}O$ was established from the MS (EI^+) M^+ at *m/z* 222.36. ^{1}H-NMR ($CDCl_3$, 300 MHz) δ 0.13 (1H, t, J = 9,2 Hz), 0.64 (1H, ddd, *J*= 12, 9,6 Hz), 0.93 (3H, d, J= 6,6 Hz), 1.01 (3H, s), 1.04 (3H, s), 1.18 (3H, s), 1.22-1.98 (12H, m). ^{13}C-NMR ($CDCl_3$, 75 MHz) δ 74.6 (C-10), 58.2 (C-1), 39.7 (C-5), 38.5 (C-4), 37.8 (C-9), 32.1 (C-14), 29.1 (C-3), 28.7 (C-12), 28.6 (C-7), 25.8 (C-2), 22.3 (C-6), 18.8 (C-8), 18.4 (C-11), 16.3 (C-15), 16.1 (C-13).

Ursolic acid (3)

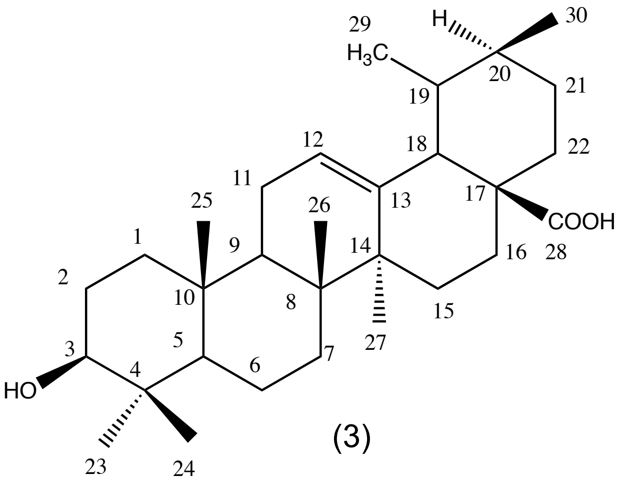

(3)

Whitish powder; mp: 210-221°C, $[\alpha]^{20}_{D}$= +52.84 (*c* 0.022 in MeOH)]. The molecular formula $C_{30}H_{48}O_3$ was established from the MS (EI^+) M^+ at *m/z* 457.79 $[M+H]^+$, 479.68 $[M+Na]^+$, 935.69 $[2M+Na]^+$. ^{1}H-NMR (CD_3COCD_3, 300 MHz) δ 5.25 (H, t, J = 3.4 Hz, H-12), 2.91 (H, m, H-3), 2.09 (H, d, J = 11.6 Hz, H-18), 1.06 (3H, s, Me-27), 0.95 (3H, s, Me-23), 0.92 (3H, s, Me-26) 0.89 (3H, d, J = 6 Hz, H-30), 0.84 (3H, d, J = 6 Hz, H-29), 0.81 (3H, s, Me-24), 0.79 (3H, s, Me-25); ^{13}C-NMR (CD_3COCD_3, 75 MHz) δ 179.3 (C-28), 139. 7 (C-13), 123.4 (C-12), 78.8 (C-3), 56.6 (C-5), 54.2 (C-18), 48.9 (C-9), 48.8 (C-17), 42.6 (C-14), 39.8 (C-8), 39.7 (C-4), 38.2 (C-19), 38.1 (C-20), 37.9 (C-1), 37.0 (C-10), 36.8 (C 22), 31.6 (C-7), 29.6 (C-21), 29.1 (C-15), 28.0 (C-23), 27.4 (C-2), 24.3 (C-16), 23.7 (C 27), 23.3 (C-11), 21.5 (C-30), 18.4 (C-6), 17.5 (C-29), 17.4 (C-26), 16.7 (C-24), 16.2 (C-25).

Oleanolic acid (4)

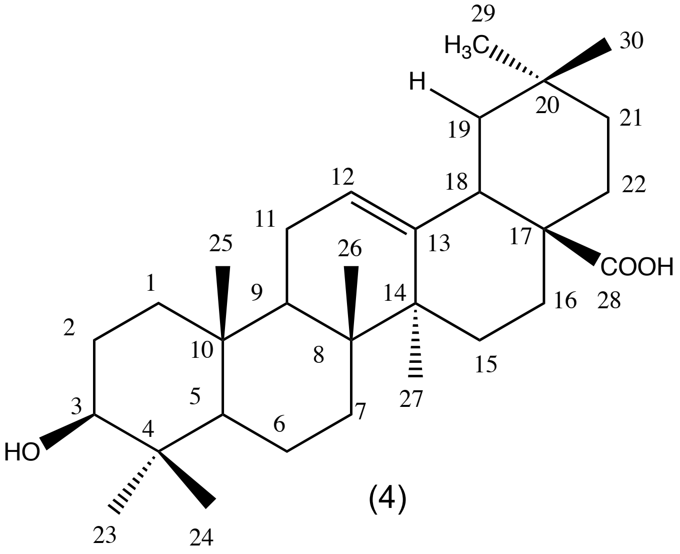

(4)

Whitish powder; mp: >300ºC, $[\alpha]^{20}_{D}$= +56.53 (*c* 0.032 in MeOH)]. The molecular formula $C_{30}H_{48}O_3$ was established from the MS (EI^+) M^+ at *m/z* 457.92 $[M+H]^+$, 479.63 $[M+Na]^+$, 935.66 $[2M+Na]^+$. ^{1}H-NMR (CD_3OD, 300 MHz) δ 5.24 (H, t, H-12), 2.98 (H, t, H-3), 2.78 (H, dd, J = 6.8, 4 Hz, H-18), 0.98 (3H, s, Me-27), 0.89 (3H, s, Me-25), 0.86 (2×3H, s, Me-30, 24), 0.83 (3H, s, H-29), 0.79 (3H, s, Me-26), 0.65 (3H, s, Me-23); ^{13}C-NMR (CD_3OD, 75 MHz) δ 181.8 (C-28), 145.4 (C-13), 123.8 (C-12), 79.9 (C-3), 56.9 (C-5), 48.9 (C-9), 48.5 (C 19), 43.4 (C-17), 40.9 (C-14), 40.6 (C-18), 40.2 (C-8), 39.9 (C-4), 38.3 (C-1), 37.0 (C-10), 33.8 (C 21), 33.3 (C-29), 32.9 (C-22), 32.5 (C-7), 30.8 (C-20), 28.7 (C-23), 27.6 (C-15), 27.4 (C-2), 26.0 (C-27), 23.8 (C-30), 23.3 (C-16), 23.1 (C-11), 17.9 (C-6), 17.8 (C-26), 16.5 (C-24), 16.1 (C-25).

5-Hydroxy-4’,7-dimethoxyflavone (5)

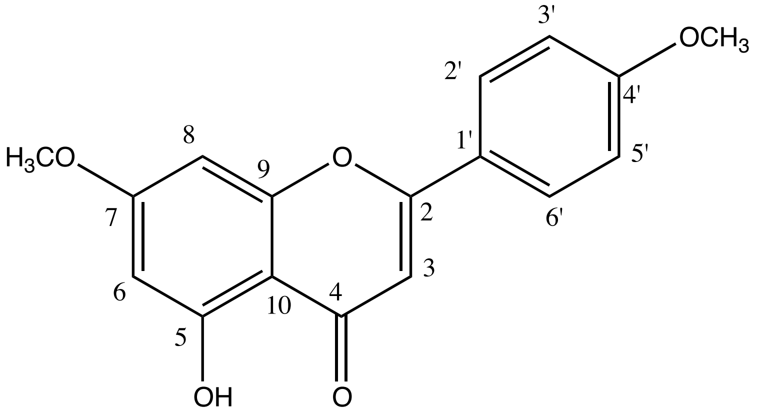

(5)

Yellow powder, mp: 216-218 ºC. The molecular formula $C_{17}H_{14}O_5$ was established from the MS (EI^+) M^+ at *m/z* 298.08. ^{1}H-NMR ($CDCl_3$, 400 MHz) δ 12.81 (1H, s, OH-5), 7.84 (2H, d, *J*= 8 Hz, H-2’,6’), 7.01 (2H, d, *J*= 8 Hz, H-3’,5’), 6.57 (1H, s, H-3), 6.48 (1H, d, *J*= 2.2 Hz, H-6), 6.36 (1H, d, *J*= 4 Hz, H-8), 3.89 (3H, s), 3.88 (3H, s). ^{13}C-NMR ($CDCl_3$, 100 MHz) δ ppm: 182.5 (C-4), 165.6 (C-7), 164.2 (C-2), 162.7 (C-5), 162.3 (C-4’), 157.8 (C-9), 128.2 (C-2’,6’), 123. 7 (C-1’), 114.6 (C-3’,5’), 105.7 (C-10), 104.4 (C-3), 98.2 (C-6), 92.8 (C-8), 55.9 (7-OMe), 55. 7 (4’-OMe).

Chrysothol (6)

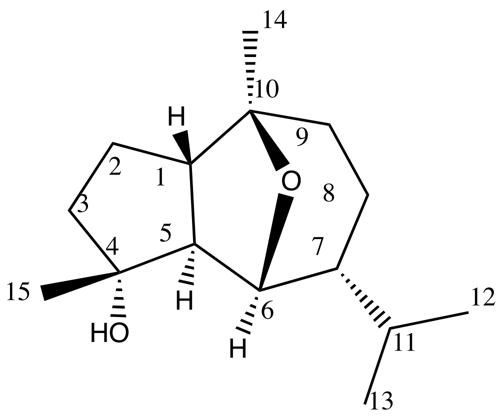

(6)

Colorless oil. The molecular formula $C_{15}H_{26}O_2$ was established from the MS (EI^+) M^+ at *m/z* 238.20. ^{1}H-NMR ($CDCl_3$, 400 MHz) δ 3.94 (1H, d, J = 4,0 Hz), 2.26 (2H, m), 2.07 (2H, m), 1.68-1.48 (7H, m), 1.34 (2H, m), 1.35 (3H, s), 1.12 (3H, s), 0.87 (2×3H, d, *J*= 7.0 Hz m). ^{13}C-NMR ($CDCl_3$, 100 MHz) δ 76.1 (C-6), 74.7 (C-4), 74.6 (C-10), 68.2 (C-5), 53.4 (C-1), 48.3 (C-3), 38.7 (C-7), 37.' (C-9), 32.8 (C-11), 25.9 (C-15), 24.0 (C-2), 22.1 (C-14), 21.2 (C-12), 21.2 (C-13), 20.4 (C-8).

Supplementary material

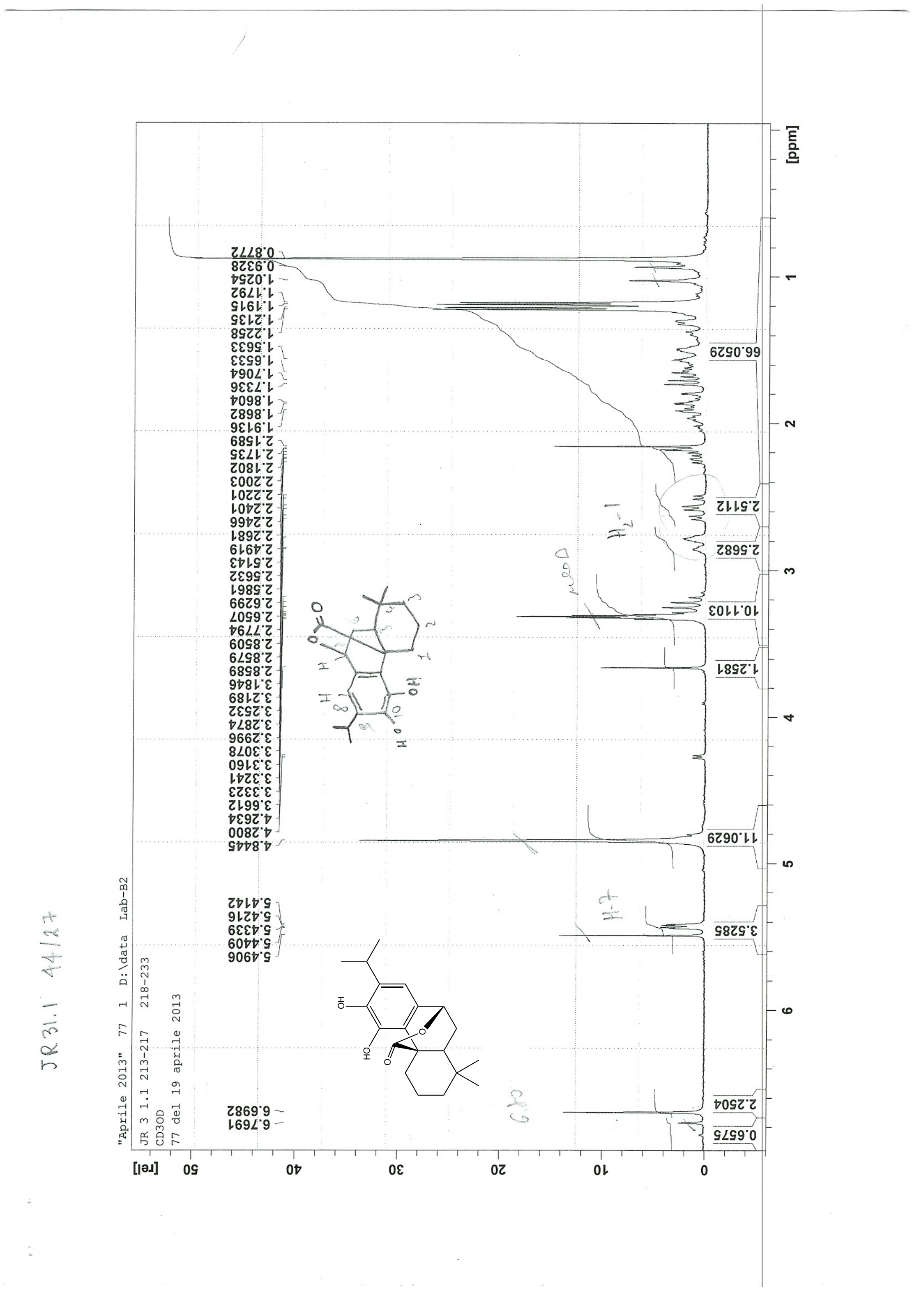

^{1}H-NMR ($CDCl_3$, 200 MHz) of carnosol.

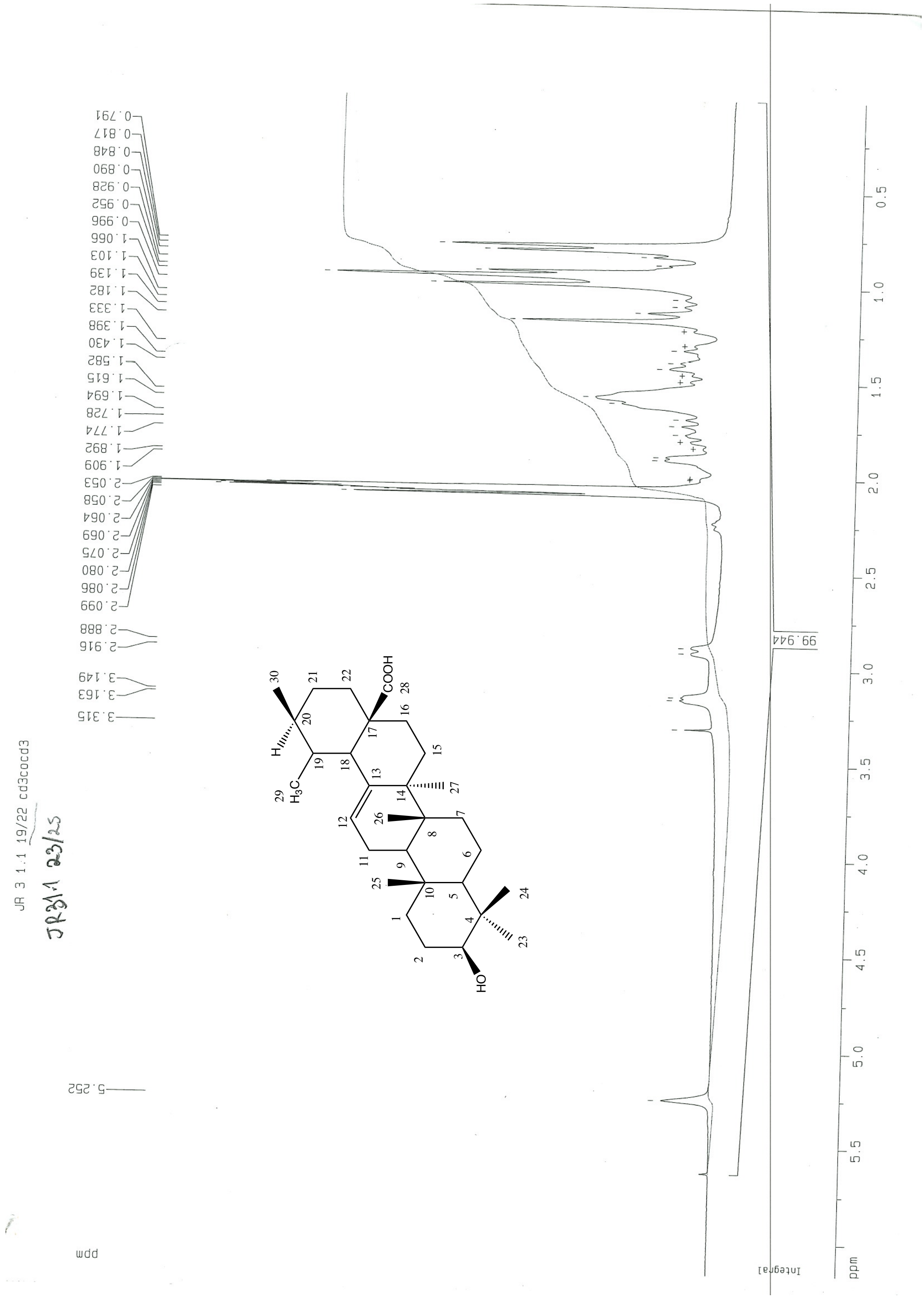

^{1}H-NMR of ursolic acid (CD_3COCD_3, 300 MHz)

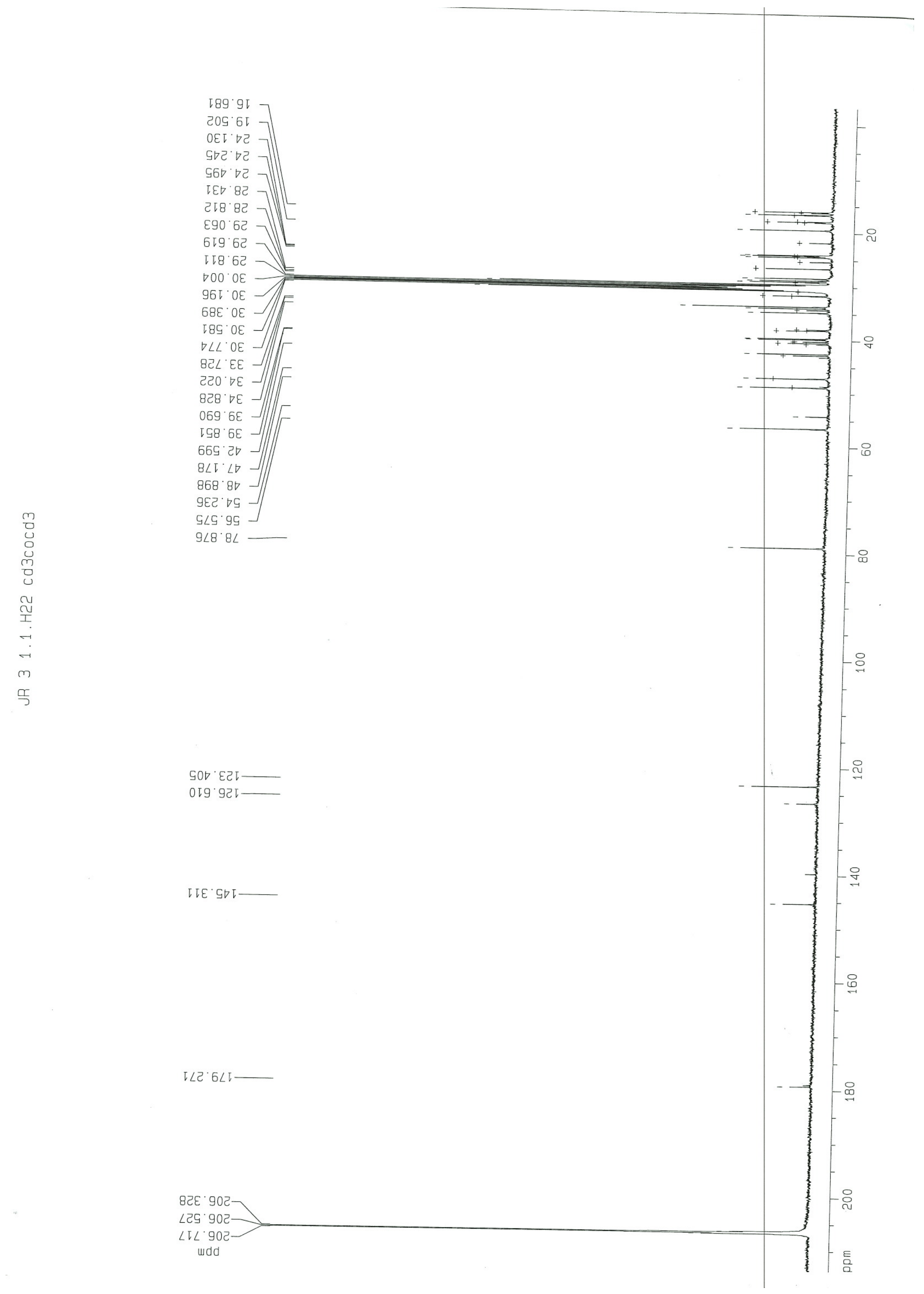

^{13}C-NMR of ursolic acid (CD_3COCD_3, 75 MHz)

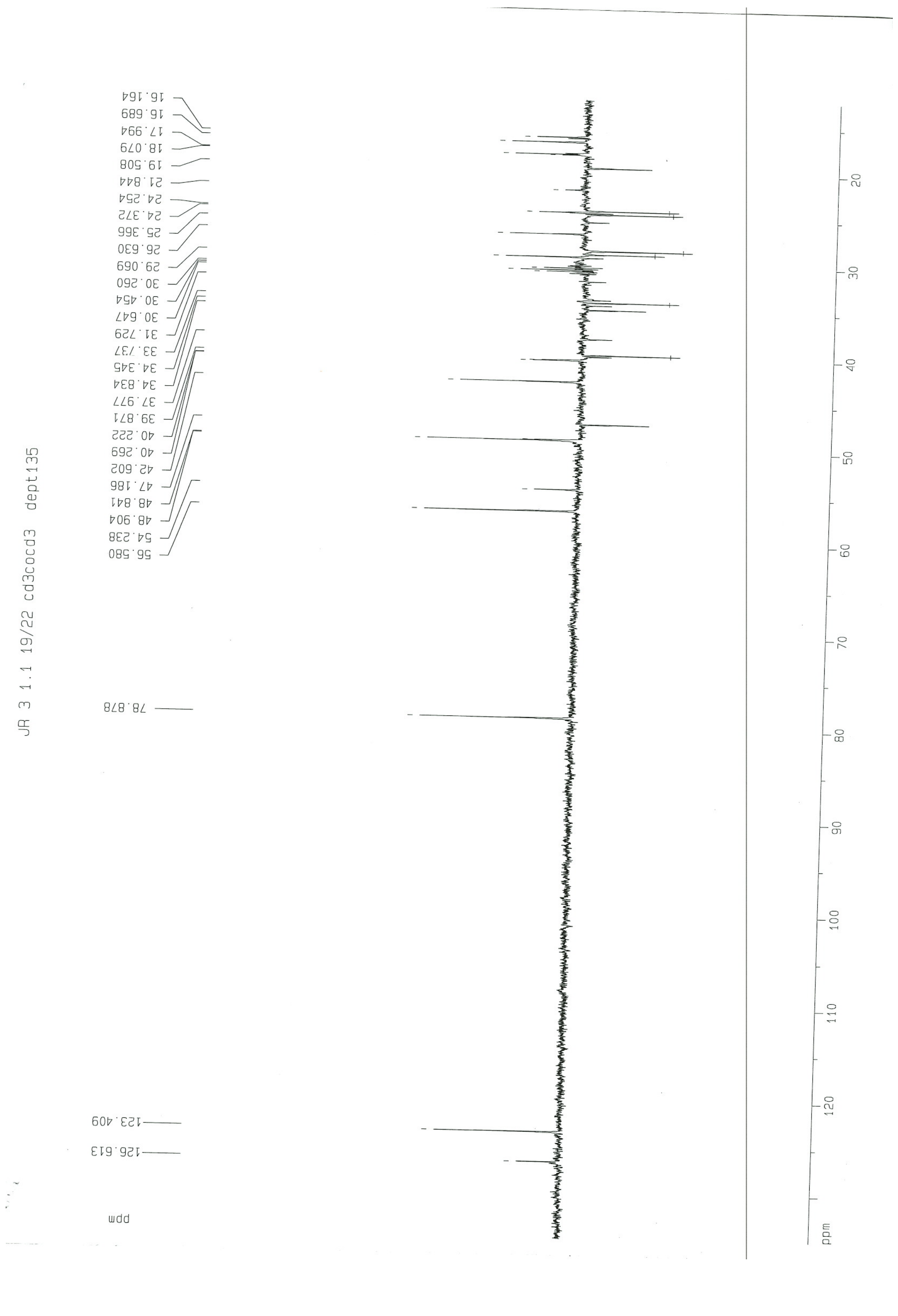

DEPT experiment (300 MHz) of ursolic acid in CD_3COCD_3.

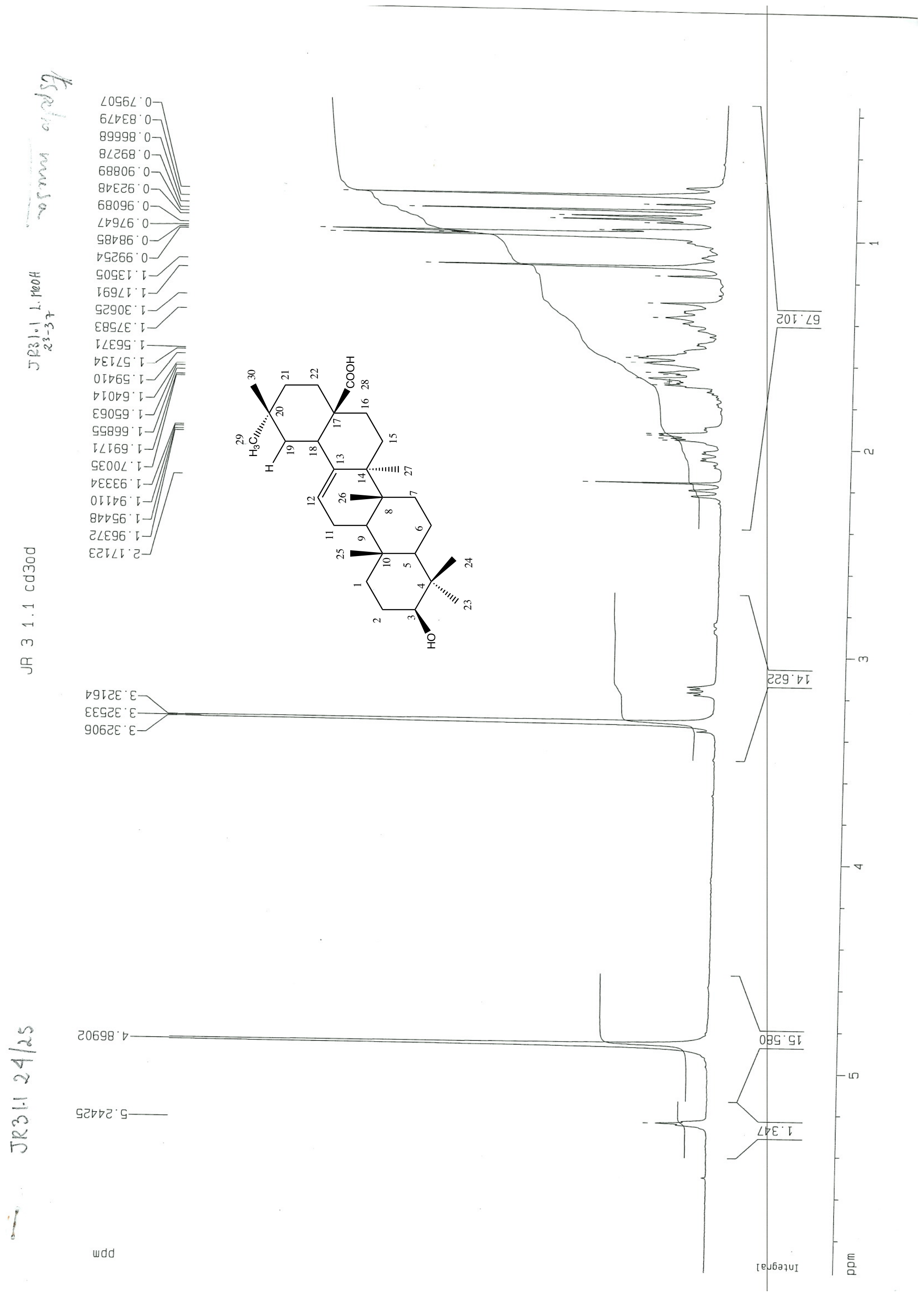

^{1}H-NMR of oleanolic acid (CD_3OD, 300 MHz)

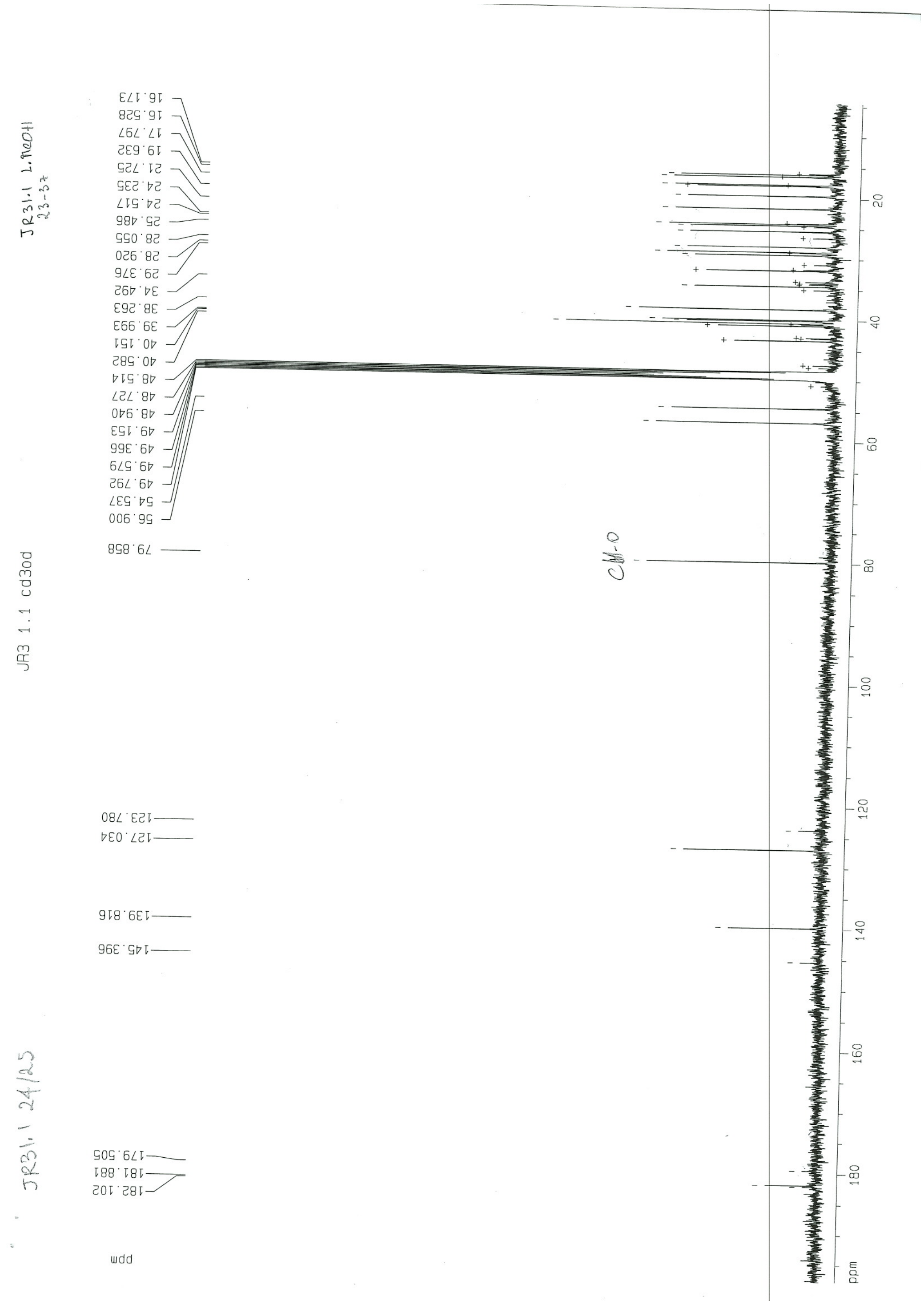

^{13}C-NMR of oleanolic acid (CD_3OD, 75 MHz)

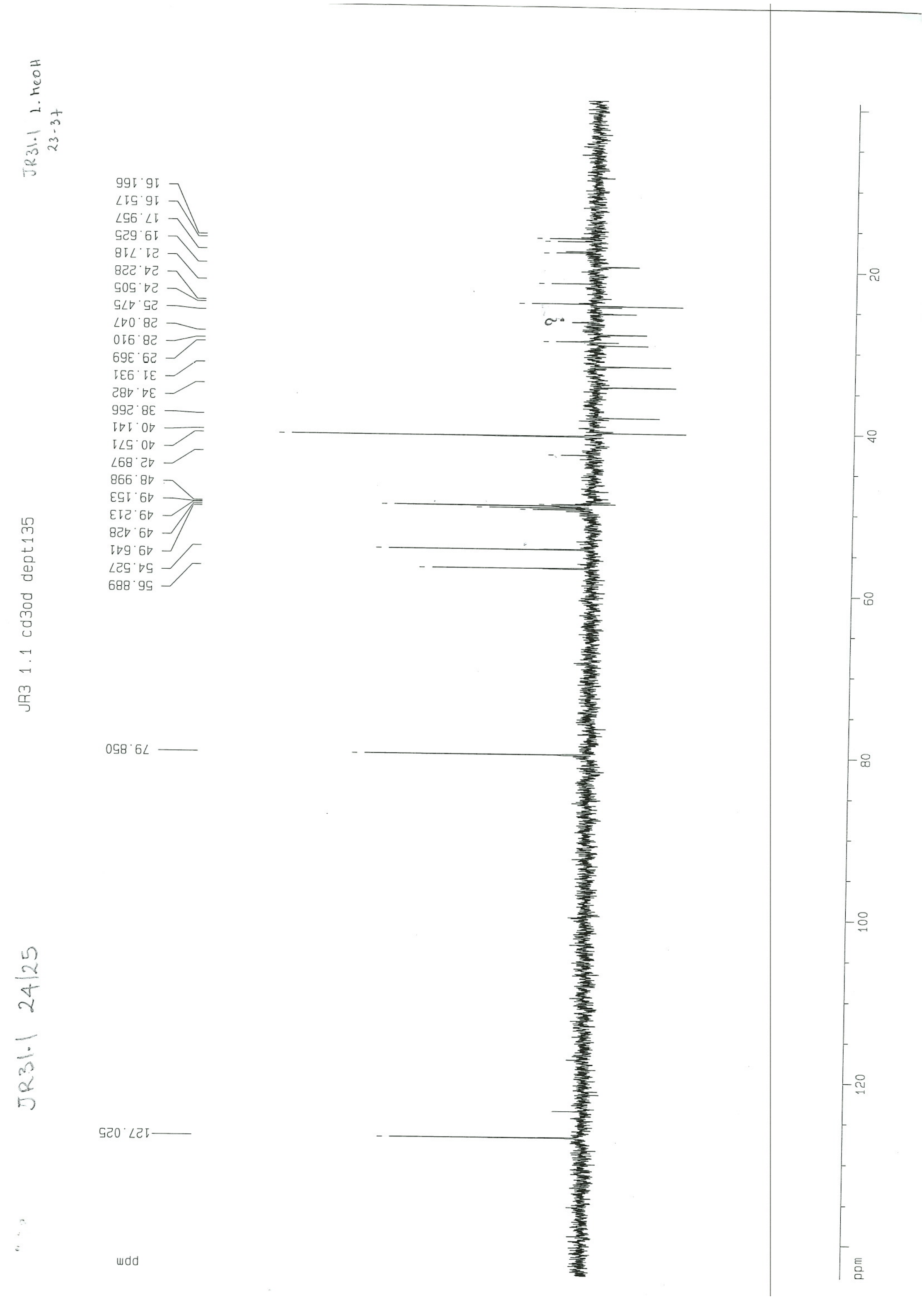

DEPT experiment (300 MHz) of oleanolic acid in CD_3OD.

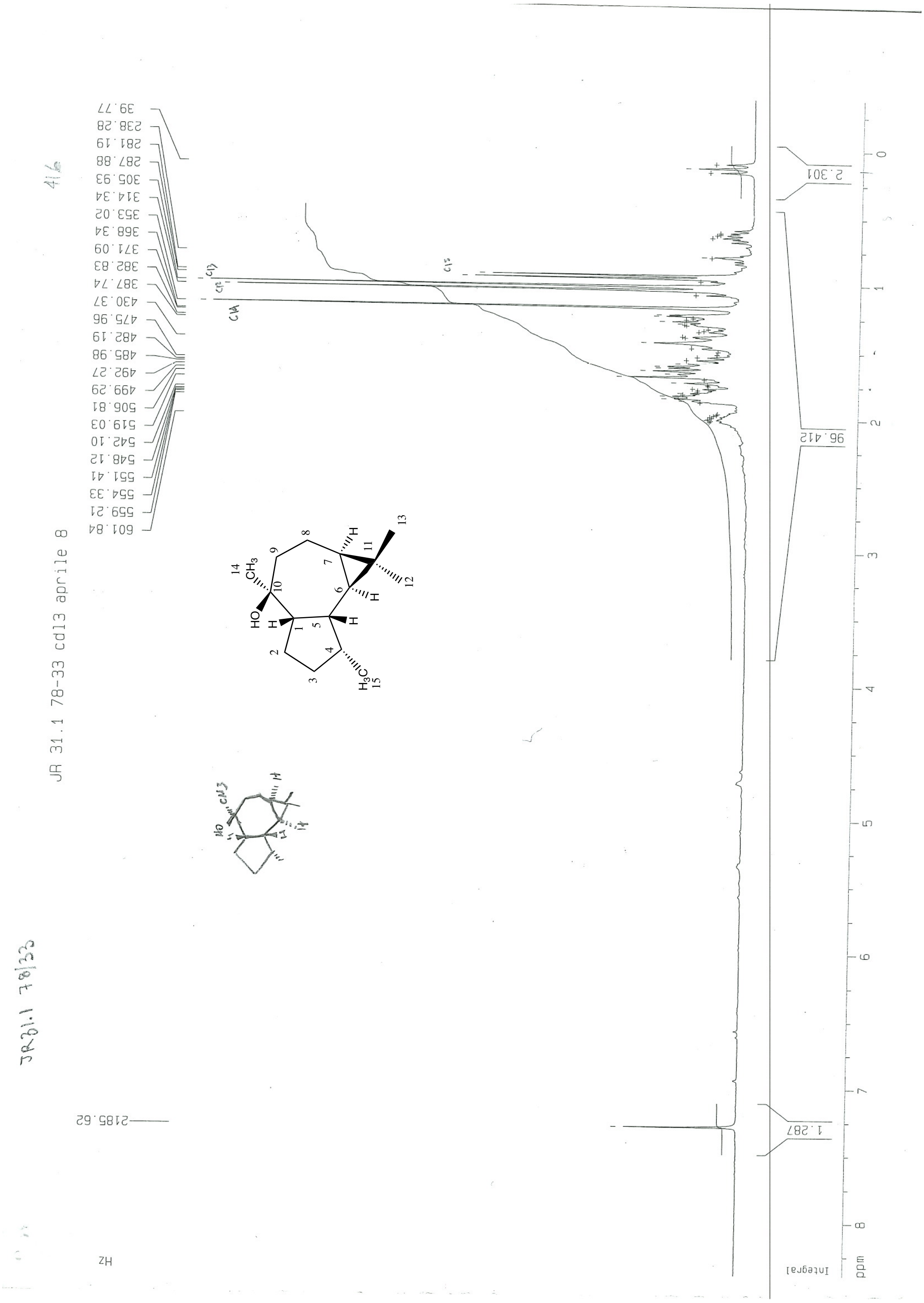

^{1}H-NMR of viridiflorol ($CDCl_3$, 300 MHz)

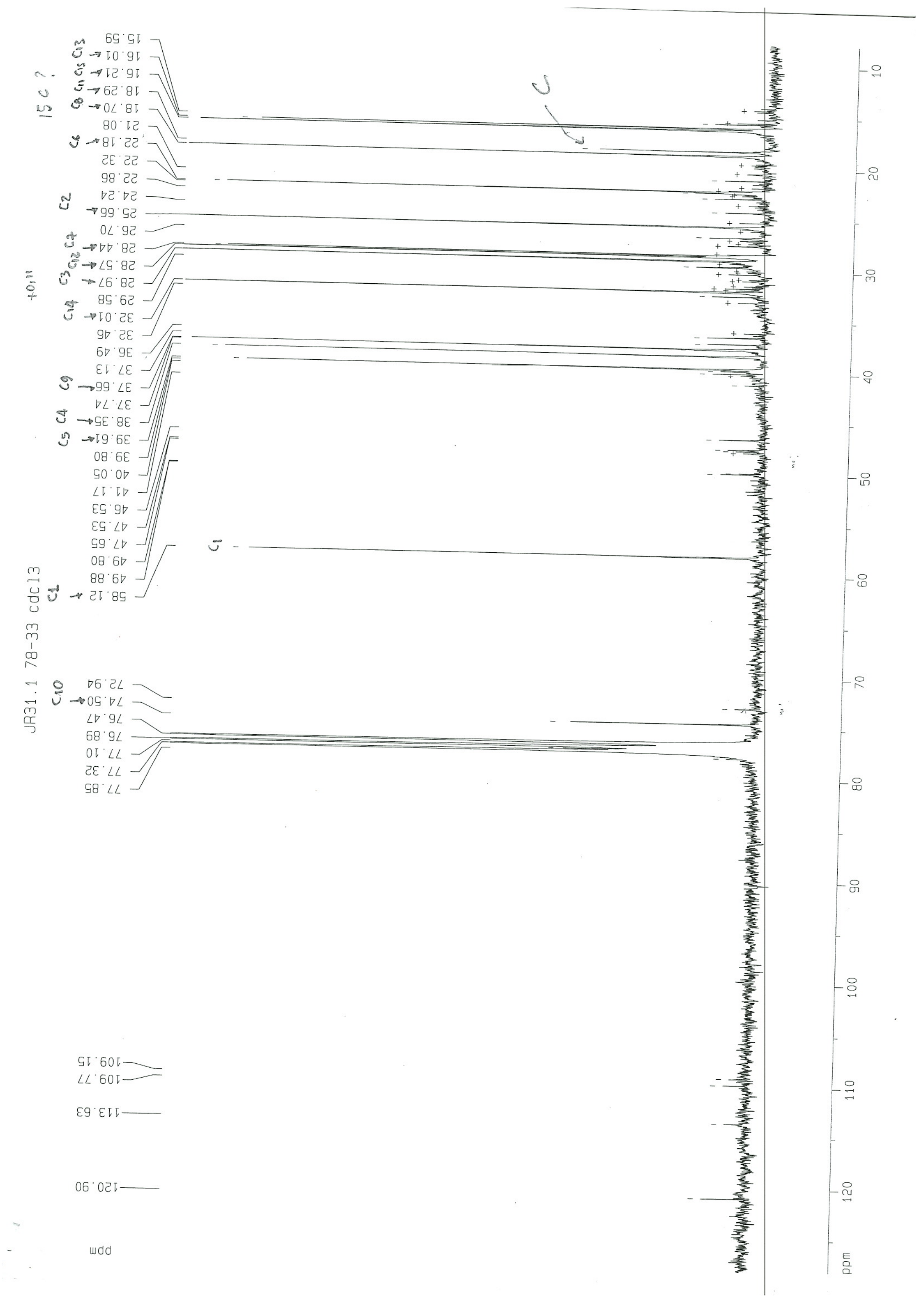

^{13}C-NMR of viridiflorol ($CDCl_3$, 75 MHz)

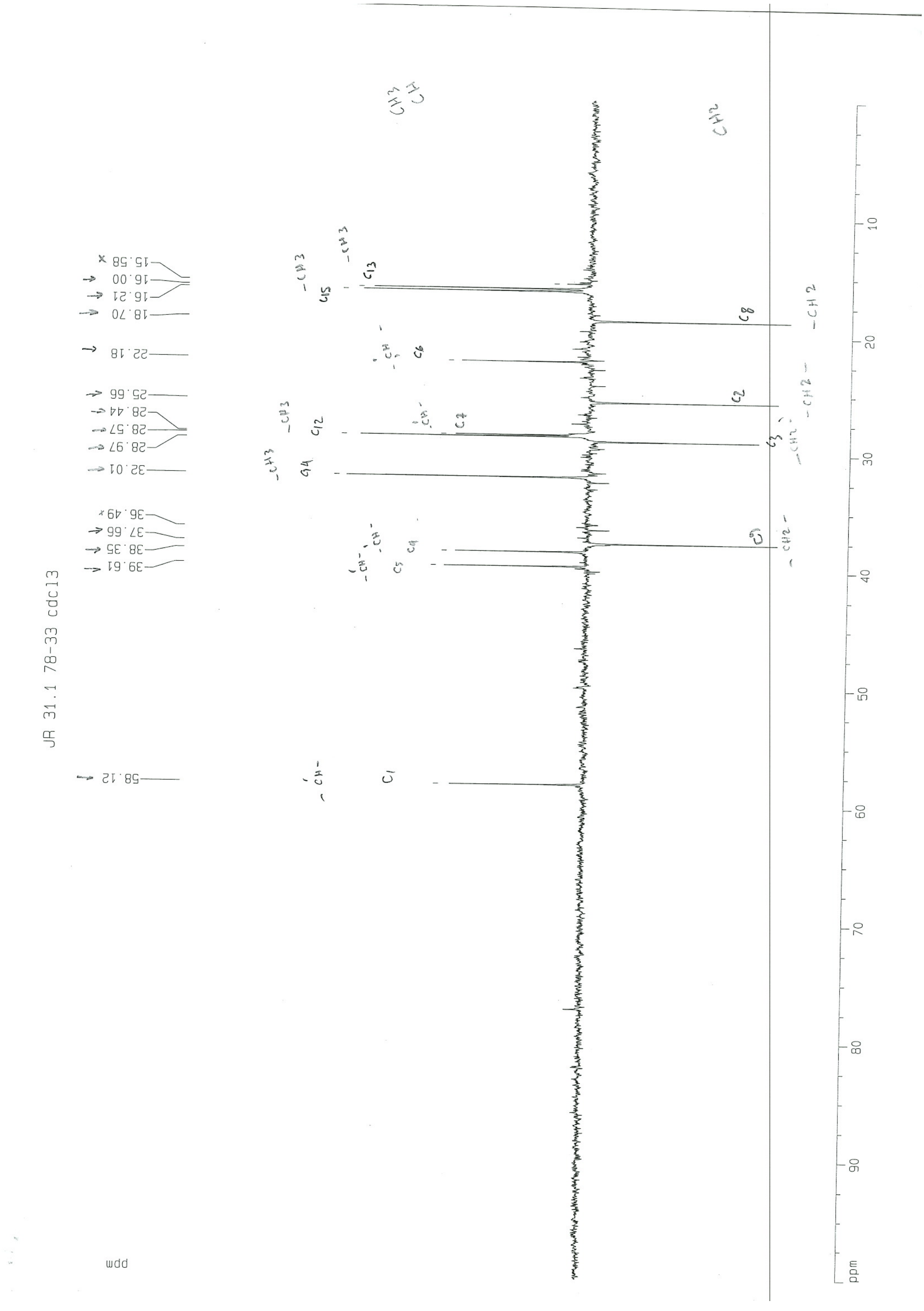

DEPT experiment (300 MHz) of viridiflorol in $CDCl_3$

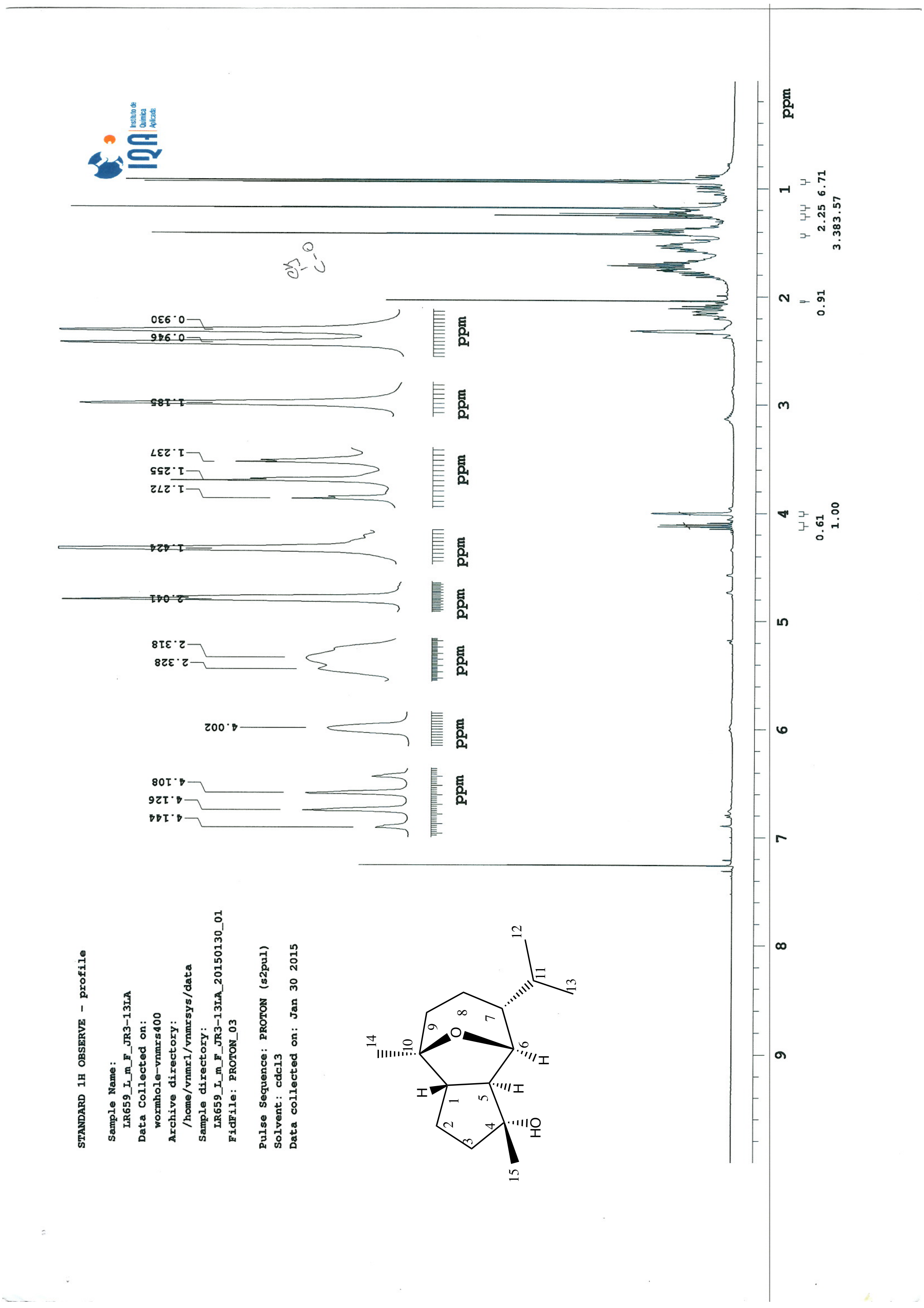

^{1}H-NMR of chrysothol ($CDCl_3$ 400 MHz)

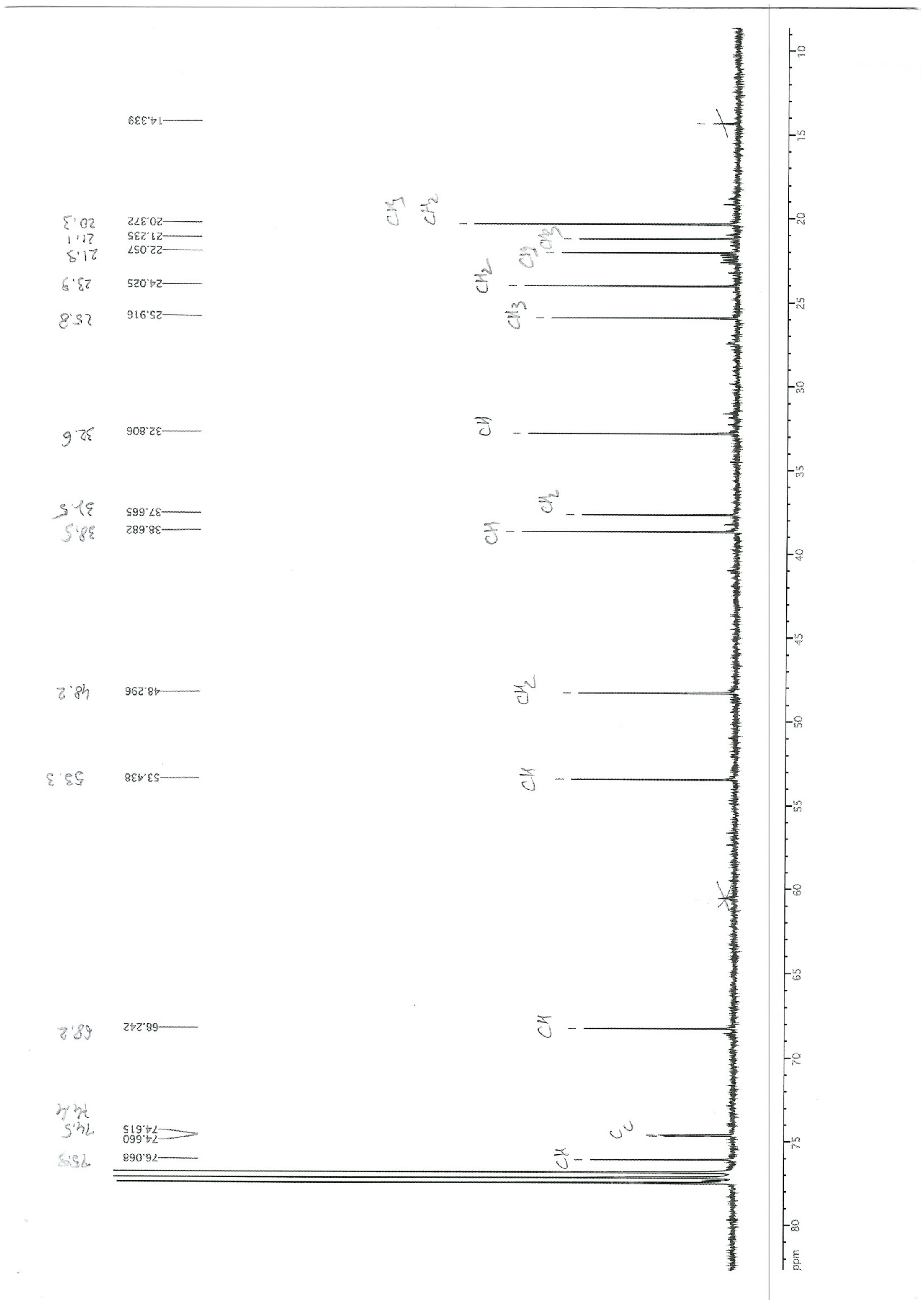

^{13}C-NMR of chrysothol ($CDCl_3$ 100 MHz)

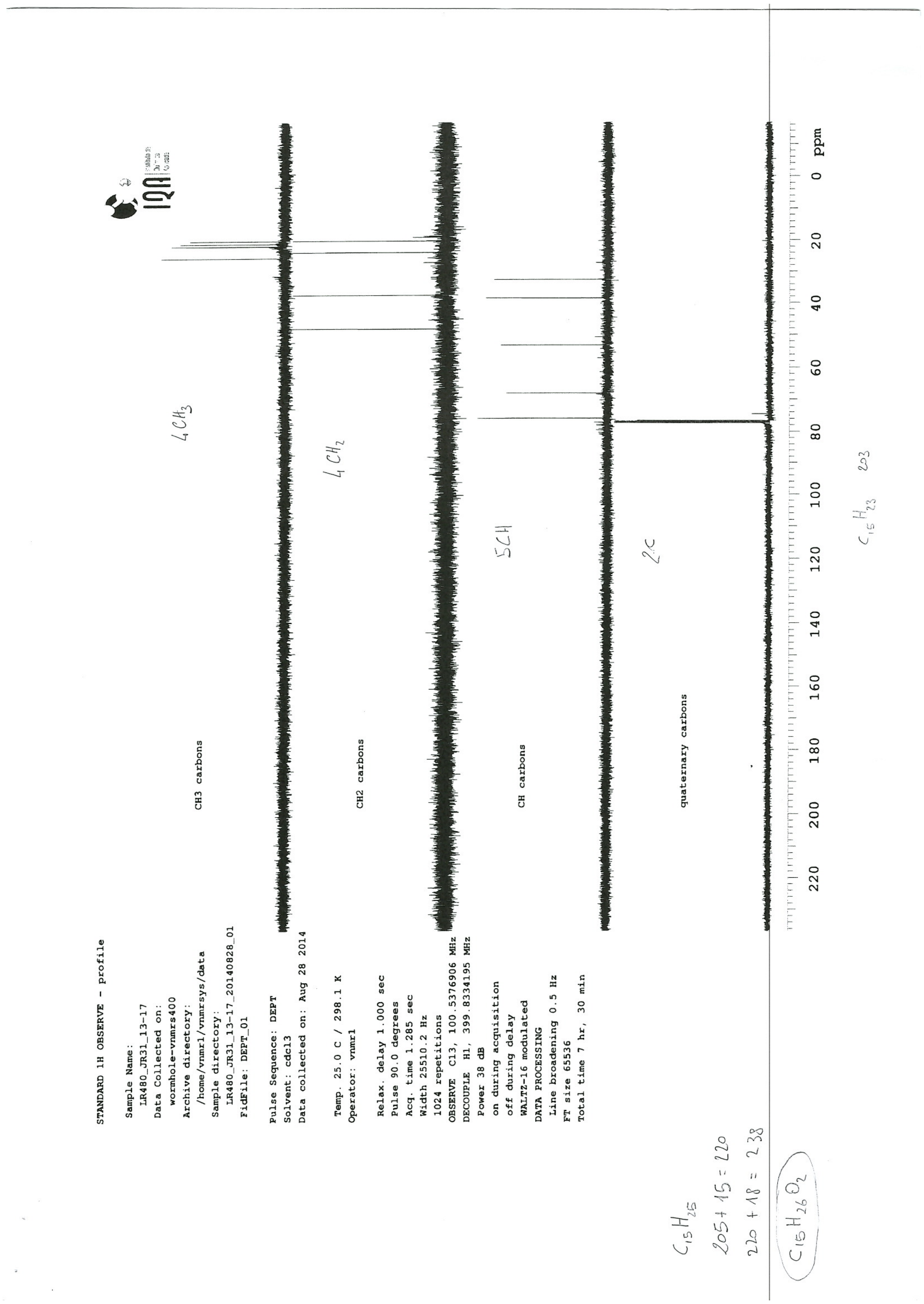

DEPT experiment (400 MHz) of chrysothol in $CDCl_3$

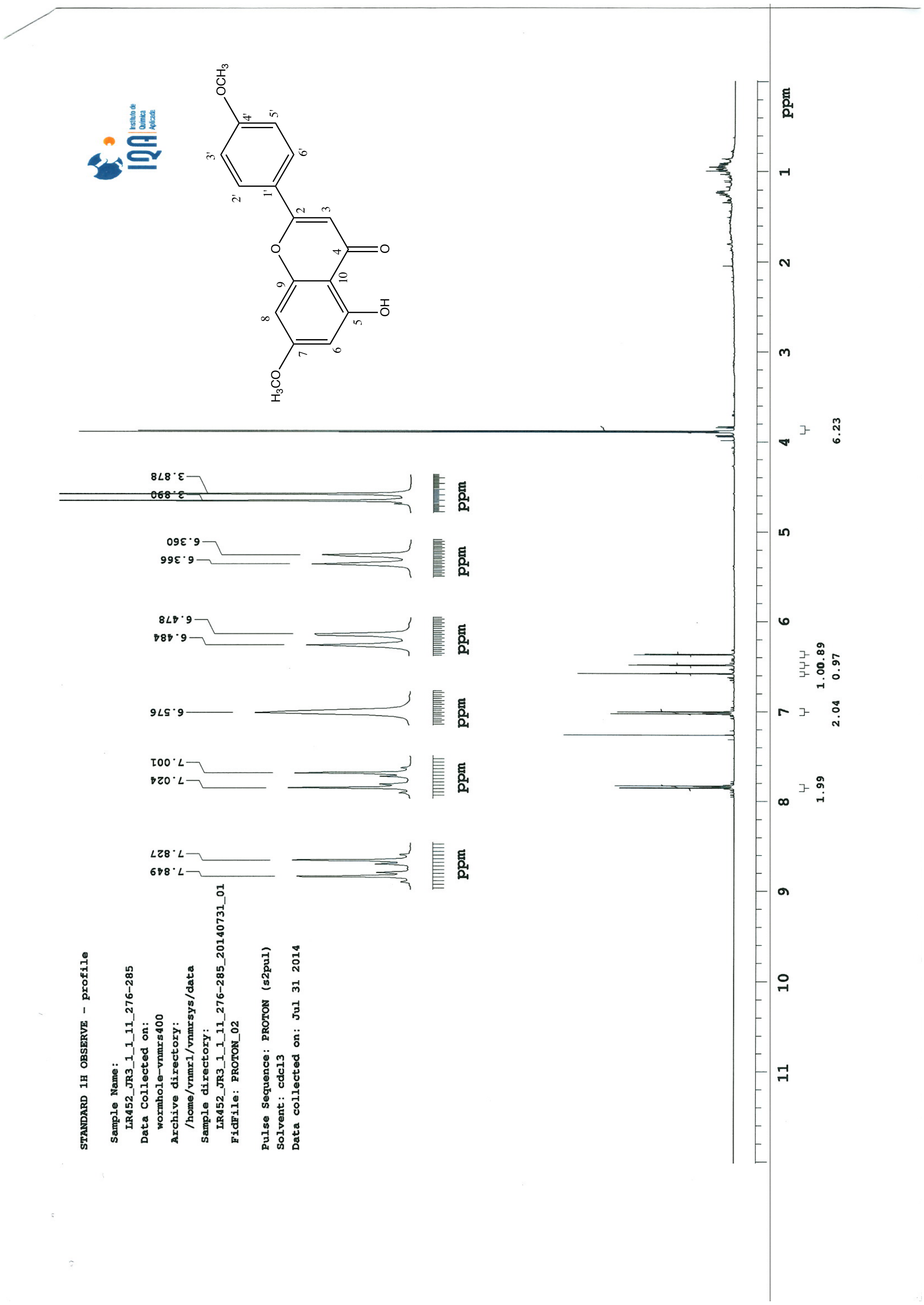

^{1}H-NMR of 5-hydroxy-4’,7-dimethoxy flavone ($CDCl_3$, 400 MHz)

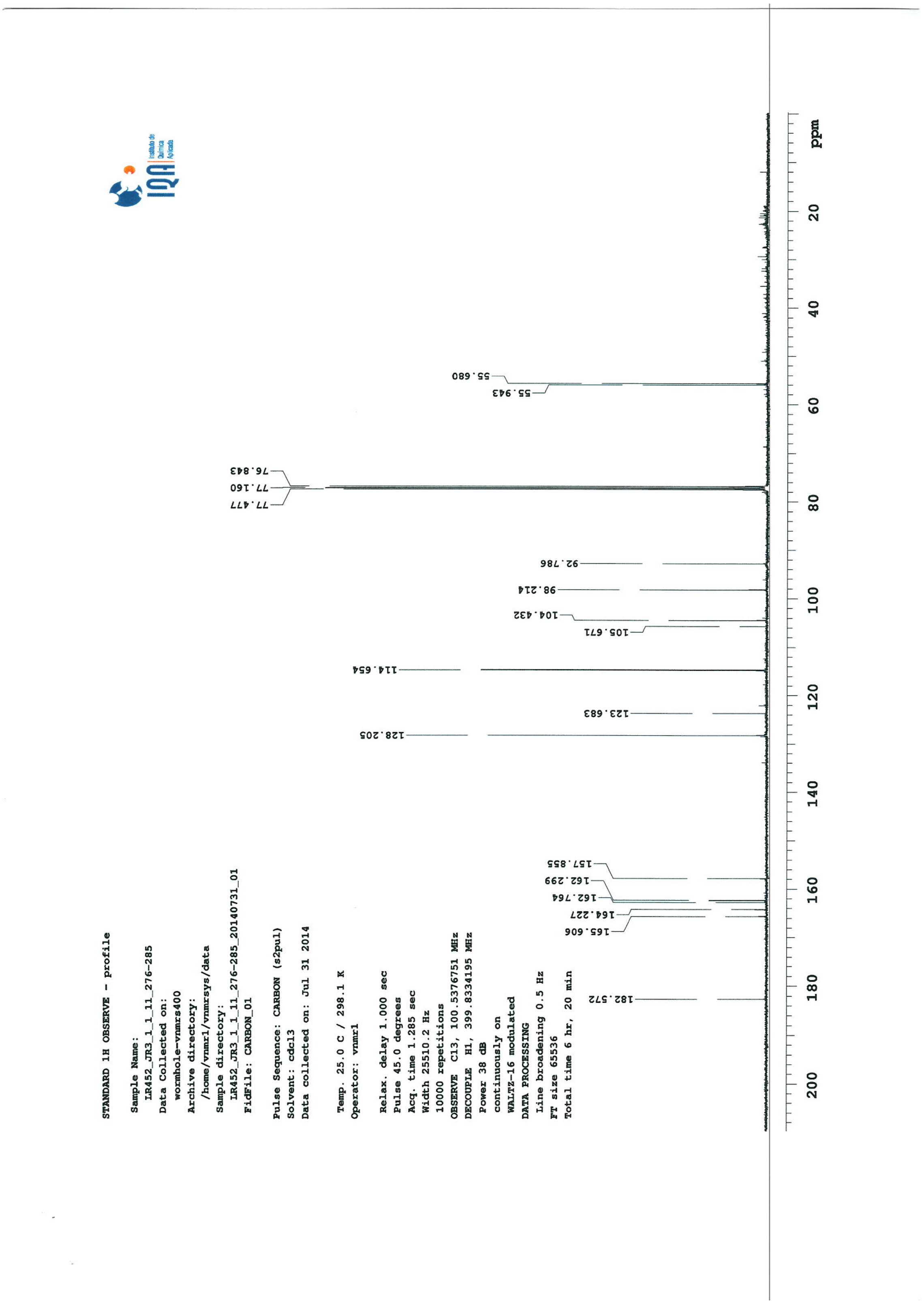

^{13}C-NMR of 5-hydroxy-4',7-dimethoxy flavone ($CDCl_3$, 100 MHz)

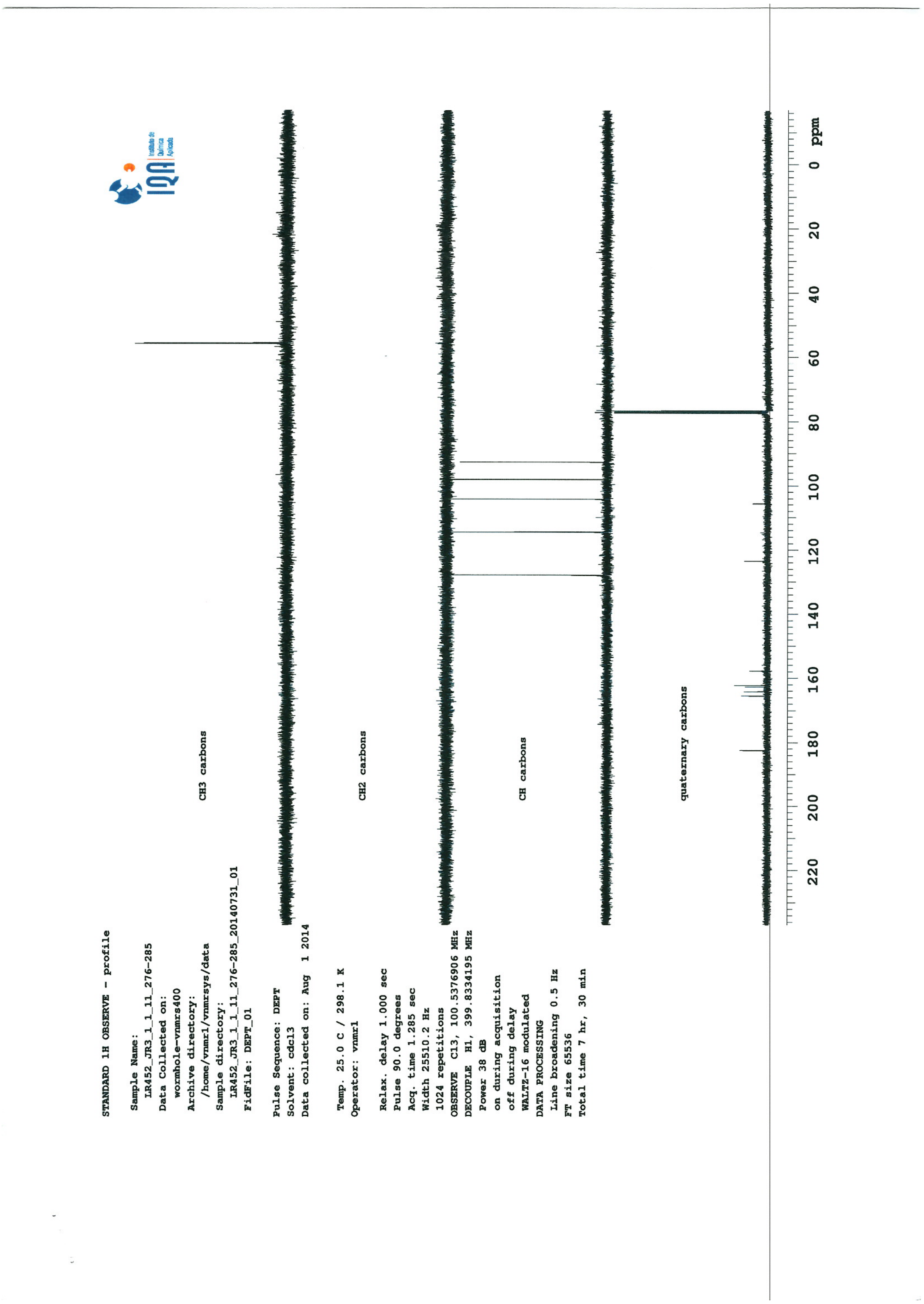

DEPT experiment (400 MHz) of 5-hydroxy-4’,7-dimethoxy flavone in $CDCl_3$
